# The spatial scaling of food web structure across European biogeographical regions

**DOI:** 10.1111/ecog.05229

**Published:** 2021-02-25

**Authors:** Núria Galiana, Ceres Barros, João Braga, Gentile Francesco Ficetola, Luigi Maiorano, Wilfried Thuiller, José M. Montoya, Miguel Lurgi

**Affiliations:** Centre for Biodiversity Modelling and Theory, Theoretical and Experimental Ecology Station, CNRS and Paul Sabatier Univ., Moulis, France; Univ. Grenoble Alpes, CNRS, LECA (Laboratoire d’Écologie Alpine), Grenoble, France; Dept of Forest Resources Management, Faculty of Forestry, Univ. of British Columbia, Vancouver, BC, Canada; Univ. Grenoble Alpes, CNRS, LECA (Laboratoire d’Écologie Alpine), Grenoble, France; Univ. Grenoble Alpes, CNRS, LECA (Laboratoire d’Écologie Alpine), Grenoble, France; Dept of Environmental Sciences and Policy, Univ. degli Studi di Milano, Via Celoria; Dept of Biology and Biotechnologies ‘Charles Darwin’, Univ. di Roma ‘La Sapienza’, Roma, Italia; Univ. Grenoble Alpes, CNRS, LECA (Laboratoire d’Écologie Alpine), Grenoble, France; Centre for Biodiversity Modelling and Theory, Theoretical and Experimental Ecology Station, CNRS and Paul Sabatier Univ., Moulis, France; Centre for Biodiversity Modelling and Theory, Theoretical and Experimental Ecology Station, CNRS and Paul Sabatier Univ., Moulis, France; Dept of Biosciences, Swansea Univ., Swansea, UK

**Keywords:** food webs, geographical variation, metaweb, network–area relationship, spatial scale, species–area relationship, terrestrial vertebrate communities

## Abstract

The species–area relationship (SAR) is one of the most well-established scaling patterns in ecology. Its implications for understanding how communities change across spatial gradients are numerous, including the effects of habitat loss on biodiversity. However, ecological communities are not mere collections of species. They are the result of interactions between these species forming complex networks that tie them together. Should we aim to grasp the spatial scaling of biodiversity as a whole, it is fundamental to understand the changes in the structure of interaction networks with area. In spite of a few empirical and theoretical studies that address this challenge, we still do not know much about how network structure changes with area, or what are the main environmental drivers of these changes. Here, using the meta-network of potential interactions between all terrestrial vertebrates in Europe (1140 species and 67 201 feeding interactions), we analysed network–area relationships (NARs) that summarize how network properties scale with area. We do this across ten biogeographical regions, which differ in environmental characteristics. We found that the spatial scaling of network complexity strongly varied across biogeographical regions. However, once the variation in SARs was accounted for, differences in the shape of NARs vanished. On the other hand, the proportion of species across trophic levels remained remarkably constant across biogeographical regions and spatial scales, despite the great variation in species richness. Spatial variation in mean annual temperature and habitat clustering were the main environmental determinants of the shape of both SARs and NARs across Europe. Our results suggest new avenues in the exploration of the effects of environmental factors on the spatial scaling of biodiversity. We argue that NARs can provide new insights to analyse and understand ecological communities.

## Introduction

One of the most fundamental patterns in ecology is the increase of the total number of species as the area sampled increases (Arrhenius 1921, Rosenzweig 1995, Lawton 1999). Thus, the species–area relationship (SAR) has been established as a fundamental property of ecological systems (Schoener 1976, Lawton 1999). It is an important tool for conservation biology and landscape ecology (Brooks et al. 2002, Rybicki and Hanski 2013). Yet, ecological communities are composed not only by species co-occurring in space, but also by biotic interactions among them, creating interaction networks. To understand the spatial scaling of biodiversity as a whole we must consider changes in the structure of species interaction networks as we change the spatial scale of observation, and the potential mechanisms behind these changes (Brose et al. 2004, Wood et al. 2015, Galiana et al. 2018).

Several mechanisms have been proposed to explain the rate of species accumulation across spatial scales (i.e. the slope of SARs). The increase of habitat diversity as area increases allows sustaining larger diversity of species with different requirements (Boecklen 1986, Drakare et al. 2006, Shen et al. 2009), while the inclusion of multiple biogeographical regions that hold species with different evolutionary histories also contributes to more species-rich communities (Drakare et al. 2006, Guilhaumon et al. 2008, Mazel et al. 2014). Mechanisms affecting the spatial scaling of the structure of interaction networks, however, need further exploration. Brose et al. (2004) proposed the scaling of the number of links with species richness as a driver of the links–area relationship. If the number of species increases with area (i.e. SARs) and the number of links per species changes as species richness increases (i.e. link–species scaling; Cohen and Briand 1984, Martinez 1992), a links–area relationship should emerge, the slope of which depends on the exponents of both SARs and the link–species scaling relationships (Brose et al. 2004). More recently, Galiana et al. (2018) presented a theoretical framework predicting the existence of multiple network–area relationships (NARs) arising from different spatial mechanisms, such as the existence of different SARs across trophic levels, the higher prevalence of generalist species at small spatial extents due to their higher probability of finding a resource, or the effects of dispersal limitation on species spatial turnover. These findings suggest that we should expect network properties to change differently with area size depending on how environmental factors (e.g. habitat heterogeneity) affect, for instance, species’ dispersal capabilities. We therefore need comparative studies focused on the variation of the spatial scaling of network structure across systems with different environmental conditions if we are to disentangle the mechanisms behind the slopes of NARs.

Although the study of the spatial scaling of network structure has progressed theoretically (Brose et al. 2004, Pillai et al. 2011, Galiana et al. 2018), empirical approaches are largely missing (but see Wood et al. 2015, Dáttilo et al. 2019, Emer et al. 2019). One exception is the work by Wood et al. (2015) on the effects of sampling and spatial scale on intertidal marine food webs in Alaska. They found that most changes in network structure across spatial scales were related to changes in species richness and connectance (i.e. the fraction of potential interactions realised). This observation is supported by further theoretical and empirical studies, which have demonstrated that many network structural properties change as species richness and connectance change, regardless of changes in area (Bengtsson 1994, Vermaat et al. 2009, Dunne et al. 2013, Eklöf et al. 2013). Yet, the question remains as to whether other factors might influence changes in the structure of species interaction networks when area size increases, independently from changes in species richness and connectance.

Due to the difficulties involved in collecting data on ecological interactions, documenting changes in network structure across spatial scales empirically is challenging: it is costly and time-consuming (Morales-Castilla et al. 2015), especially if one attempts to analyse vast spatial extents, as it has been done for SARs. To overcome this challenge, different approaches infer species interactions that complement information on observed ones, based on expert knowledge, literature reviews on who interacts with whom, or proxies such as species traits (Gravel et al. 2013, Albouy et al. 2014, Morales-Castilla et al. 2015). This generates a network of potential interactions (or ‘metaweb’ sensu Dunne, 2006) that captures all potential interactions between species of a given regional pool. These metawebs can be further constrained with information on species distributions, habitat preferences or environmental conditions to better characterize local assemblages (Albouy et al. 2014, 2019, Bartomeus et al. 2016, Braga et al. 2019). The use of metawebs is rising due to the increasing availability of high-quality data and the development of new analytical tools that allow better inference of the presence/absence of interactions. This opens new avenues not only to analyse network structure at large biogeographical scales, but also to capture valuable information on the processes that structure communities at different spatial scales by comparing the local assemblages with the regional metaweb. For instance, if local assemblages deviate from the metaweb in a given section of an environmental gradient, this might reflect higher levels of beta-diversity, which in turn generates more variation in species composition and in their biotic interactions across space, resulting in larger changes in network structure across spatial scales.

Here, we aim to characterise network–area relationships across an entire continent to identify the main drivers of the slopes of NARs. We combine Tetra-EU 1.0, a species-level trophic metaweb of European tetrapods (Maiorano et al. 2020), with the known distribution of these species (Maiorano et al. 2013), to determine: 1) whether different properties of network structure are equally affected by area size, 2) whether geographical variation across Europe exists in the spatial scaling of network structure, 3) what are the main environmental determinants of the variation among biogeographical regions and 4) what is the contribution of species richness to the patterns observed in NARs.

## Methods

We built species–area and network–area relationships (SARs and NARs respectively) for ten European biogeographical regions, defined by the European Environmental Agency (<www.eea.europa.eu>), that are characterised by different environmental conditions and habitat characteristics. We determined food web structure at different spatial scales by combining two sources of information to infer trophic links between species pairs A and B: 1) the co-occurrence of species A and B at the spatial scale analysed (based on species distribution maps), and 2) the existence of a potential interaction between species A and B in the metaweb as described in Maiorano et al. (2020). We then determined the specific SAR and NAR for each biogeographical region and compared the scaling exponents of SARs and NARs across the ten regions as descriptors of the rate of change of species and network structure with area. We analysed the effects of species richness on network structure to assess how accurately can NARs be mapped by their corresponding SARs. Lastly, we analysed the environmental and spatial factors determining the slope of SARs and NARs across biogeographical regions.

### Study area and species distributions

Our study area comprises the entire European subcontinent (except Macaronesia and Iceland), and western regions of Turkey and Russia. We refer to this area as Europe. Maps of each biogeographical region within Europe were obtained from the European Environmental Agency (EEA). The EEA has defined a zonation of Europe into biogeographical regions (hereafter bioregions), based on similarities in environmental conditions and habitats across these. We used 10 European bioregions from this classification: Alpine, Anatolian, Arctic, Atlantic, Black Sea, Boreal, Continental, Mediterranean, Pannonian and Steppic. A full description of each bioregion is available online (<www.eea.europa.eu>).

Species ranges (i.e. distribution maps) for terrestrial vertebrates within the study area at a 300 m resolution were obtained from Maiorano et al. (2013). We upscaled all species range maps to a 10 × 10 km equal-size area grid. Species were considered present on a given 10 × 10 km cell if they were present in at least one of the 300 × 300 m cells within it. This yielded species distributions maps for 510 species of breeding birds, 288 mammals, 239 reptiles and 103 amphibian species, which together comprise our species distribution database.

### European terrestrial vertebrate metaweb

Trophic interactions between all species in the database were taken from Tetra-EU 1.0 (Maiorano et al. 2020). This dataset comprises a continental scale, species-level, metaweb of trophic interactions (i.e. food web) connecting all tetrapods (mammals, breeding birds, reptiles, amphibians) occurring in Europe and in the northern Mediterranean basin. A trophic interaction was defined as potential predation on any life stage of a species (e.g. egg and larval when applicable, juvenile or adult). All trophic interactions described in the metaweb are qualitative (i.e. presence/absence of interaction) and are based on data extracted from scientific literature, including published papers, books and grey literature. For each species, all the potential trophic interactions with all other tetrapods in Europe were gathered. Thus, a trophic link between any given pair of species was added to the metaweb if the interaction was described in the analysed literature. Trophic interactions that were not described in the literature were considered absent. For the few species for which there was very little information on their diet or prey, the prey of the closest species in terms of phylogeny and morphological characteristics were considered as potential diet of the species. When possible, the sources of literature considered to determine all the potential interactions were focused specifically on the trophic interactions of the species measured or inferred within the study area. All tetrapod species whose diet did not include another species of the metaweb (such as herbivores, insectivores, piscivorous and detritivores) were defined as basal species (Braga et al. 2019). Maiorano et al. (2020) for a complete description of the data and specific data sources used to build the metaweb. The metaweb used in this study comprised 67 201 trophic interactions distributed across 1140 terrestrial vertebrate species (70% of basal species, 12% of which were herbivores and 58% non-herbivore basal species, 28% were intermediate species and 2% were top predator species). Table 1 shows the values of all food web properties for the metaweb.

### Local assemblages and food web properties

Local assemblages were built by intersecting the metaweb information with species distribution maps. For each 10 × 10 km cell in the map of Europe, we considered all species present and determined the interactions between them using the information provided by the metaweb. That is, for every pair of co-existing species in a 10 × 10 km cell of the map, we checked whether a trophic interaction between them exists in the metaweb. When building local networks, species sitting at the base of the food web (i.e. basal species) were considered to be those vertebrate species consuming resources such as carrion, plants, invertebrates or fish; since the latter are assumed to be present across the entire geographical range considered. Once local food webs were built using the criteria outlined above, we analysed their structure by quantifying several network properties that are commonly used in food web studies.

We calculated the following network metrics: number of species (S), number of links (L), links per species (L/S), mean and standard deviation of vulnerability (number of predators per prey) and generality (number of prey items per predator), fraction of basal (B), intermediate (I) and top (T) species (i.e. species without prey, with both prey and predators and without predators, respectively) and the proportion of consumers’ diet overlap (i.e. fraction of predatory links shared by predators) (see the Supporting information for details on how these properties are calculated). The standard deviations of generality and vulnerability quantify the respective variability of species’ prey and predator counts across species in an assemblage and, therefore, they inform about how different species are in terms of their number of prey and predators. We also quantified network modularity using the formulation proposed by Newman and Girvan (2004), but the values obtained ranged from 0 to 0.07 across all bioregions, indicating that the networks analysed showed extremely low modularity values. Therefore, we excluded modularity from the analyses (Supporting information). All network analyses were implemented in the R package *igraph* (Csardi and Nepusz 2006).

### Building network-area relationships

The three elements described above: species distributions maps, the metaweb and network properties, allowed us to build NARs. The spatial resolution of species distribution maps (i.e. 10 × 10 km) determined the local scale of our study. To simulate a spatial scale continuum, we iteratively aggregated map cells, one by one, into larger areas of different sizes (see below for a detailed explanation of the aggregation procedure). Once sampling areas were defined, we constructed food webs at each spatial scale using the information on species presence/absence for each aggregation of map cells and extracted from the metaweb the corresponding trophic interactions between the co-occurring species. Food web structure at each spatial scale must be understood as the structure of the subnetwork of the metaweb comprised by the species found in that area. In this way, we calculated food web properties at each spatial scale. This allowed us to lay out the relationships between area size (i.e. number of map cells) and network properties: the NARs.

### Spatial aggregation

**Figure 1:**
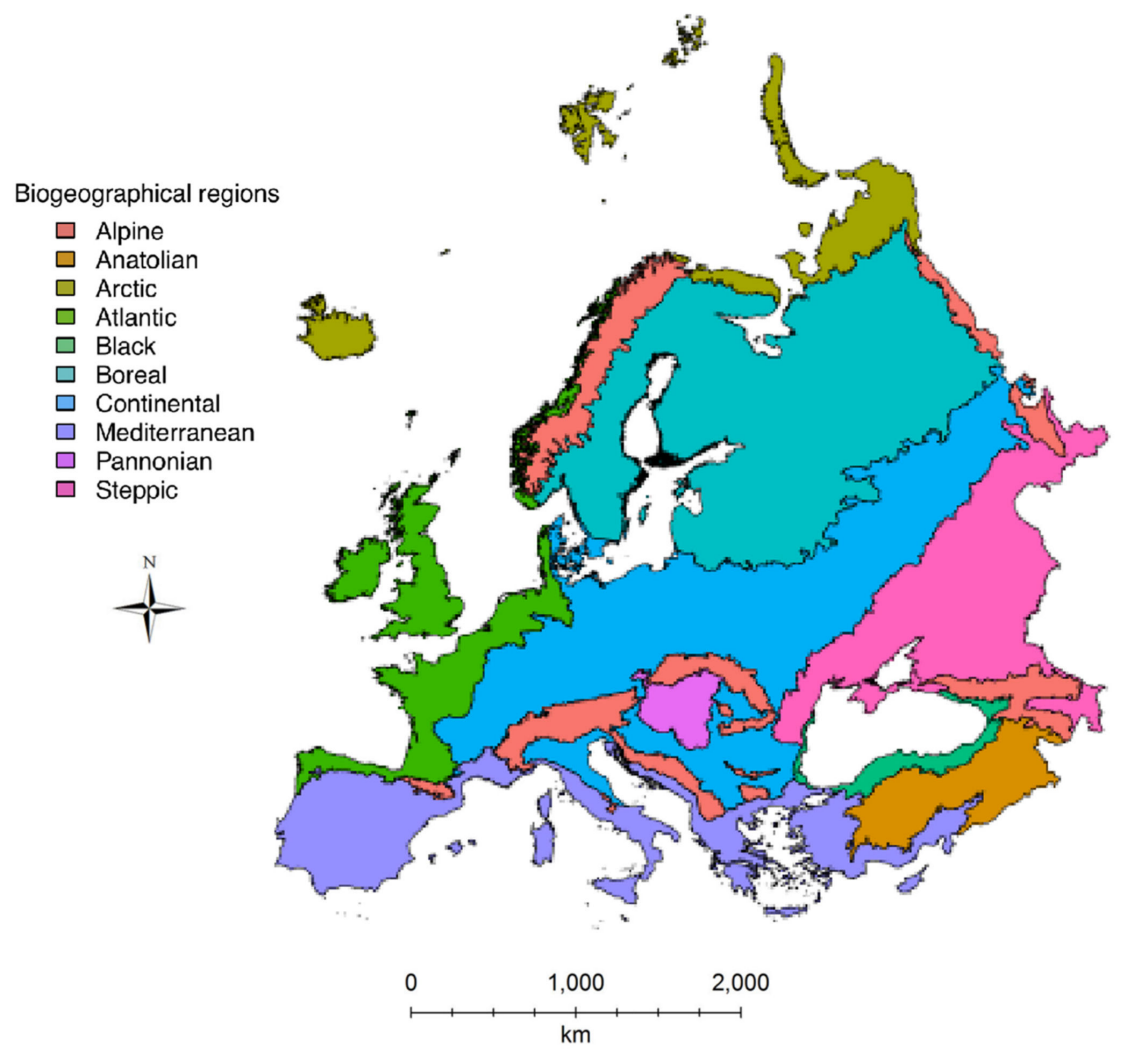
Study area. European biogeographical regions, as defined by the European Environmental Agency (<www.eea.europa.eu/data-and-maps/data/>; accessed on March 2020), considered in our analyses of the geographical variation in the spatial scaling of biodiversity.

To simulate a continuum of spatial scales, we aggregated map cells to increase the area sampled, starting from a single cell. Map cells can be aggregated in several different ways to consider larger spatial extents, such as a random aggregation across the entire range, or a linear aggregation based on nearest neighbours (Storch et al. 2008). Because ecological communities in nature comprise assemblages of species that live close to each other within a continuous spatial extent, we developed an algorithm for cell aggregation that ensures spatially coherent communities at different scales. Starting from a randomly chosen cell, our algorithm aggregates cells by choosing neighbouring ones in a ‘spiral’, ever-increasing way from the local (i.e. one 10 × 10 km cell) to the desired spatial scale. The largest (i.e. regional) spatial scale comprises the aggregation of all cells within the entire spatial extent considered, i.e. a biogeographical region. Since the starting point of this aggregation procedure is randomly chosen, species composition of communities, especially at small spatial scales, is dependent on the geographical location of this starting point. This procedure was thus repeated 100 times for each biogeographical region independently and starting from different random locations to account for the variability arising from the choice of the starting point of aggregation (i.e. the first cell). This simulation protocol produced 100 replicates of NARs and SARs for each of the biogeographical regions considered (Fig. 1). To determine bioregion membership of each cell on the species distribution maps (drawn at the whole European scale) within each bioregion, we overlaid individual maps for each bioregion (obtained from EEA as mentioned above) on the species distribution maps using the *rgdal* package in R (Bivand et al. 2018). This allowed us to build NARs and SARs independently for each bioregion.

### Species richness contribution

To assess the importance of species richness on the spatial scaling of network properties, we used three different methods: 1) linear regressions between each network property and species richness; 2) comparison with null models where we change the structure of the network while maintaining the same number of species; 3) normalising network properties by the number of species and looking at their spatial scaling.

The linear regression models between each network property and species richness allowed us to analyse the R^2^ of the models across all biogeographical regions to determine how well network properties could be predicted from species richness. This method enabled us to determine the contribution of species richness to the patterns observed while fully maintaining the structure of the network of interactions (i.e. the degree distribution of the network is conserved). On the contrary, with the null model approach, we built networks with the same number of species while breaking the original structure of the networks following two different strategies. Concretely, we generated two null models with the same principle: for each cell of the map, we checked the number of species present and we randomly picked the same number of species from the metaweb. We then built the network for those species following two different criteria. For the null model-1, we took from the metaweb all the interactions present between the selected species. We call these assemblages subsampled networks. For the null model-2, we checked the number of links present in the selected cell and randomly distributed those links between the selected species. We call these assemblages random networks. Thus, the null model-1 allowed us to determine the contribution of the identity of the species (with their respective links) to the observed patterns, while in the null model-2, given that there is no inherited structure from the metaweb, we completely broke the structure of the original network to further test whether there was any contribution of area into network structure beyond species richness. For both null models, we generated networks of different sizes by adding the number of species of subsequent cells in a similar fashion than we did for the spatial aggregation of cells. At each step of species addition, we calculated all network metrics. We replicated the procedure 100 times for each bioregion. We evaluate the resulting network–area relationships (where area is the number of cells from which we extracted the number of species) by fitting a power function. We finally compared the fitted parameters with those obtained in the original network–area relationships.

### Spatial and environmental variables

To assess whether differences in the shape of SARs and NARs across bioregions were related to their environmental features, we characterised bioregions according to different aspects of their environment and spatial complexity. Specifically, we considered the average and standard deviation of mean annual temperature, temperature seasonality, mean annual precipitation and precipitation seasonality across cells, dissimilarity (Bray–Curtis) and spatial clustering (Moran’s I) of habitat composition, total area of the bioregion and total number of habitats contained within each bioregion.

Different aspects of both temperature and precipitation have been shown to affect network structure in different systems (Dalsgaard et al. 2011, Schleuning et al. 2012, Poisot et al. 2017). We extracted values for mean annual temperature, temperature seasonality, mean annual precipitation and precipitation seasonality across Europe from WORLDCLIM ver. 2 (Fick and Hijmans 2017) using the *raster* package in *R* (Hijmans and van Etten 2014). We used the geographic resolution of 5 arc minutes provided by WORLDCLIM to match the 10 × 10 km resolution cells of the species distribution maps. Because we were interested in how network structure changes across spatial scales, we were interested in a single summary measure for each of these environmental variables across the whole spatial extent in order to relate that measure with the scaling of SARs and NARs. We calculated the mean and the standard deviation of each climatic variable across all cells within each bioregion to obtain such as measure. This allowed us to capture the effect of environmental variability across space on the changes in network structure as we increased the spatial scale of observation.

Habitat dissimilarity and spatial clustering of habitats were based on the land cover map extracted from GlobCover V2.2 (<http://due.esrin.esa.int/page_globcover.php>), which comprises 46 land-cover classes at the European level. We calculated the proportion of each land-cover class at a 300 m resolution within every single 10 km cell. Habitat dissimilarity was quantified using the Bray–Curtis dissimilarity index, which in our case quantifies the dissimilarity between two map cells based on the cover of unique habitats found within them:

(1) $$BC_{ij}=1-\frac{2C_{ij}}{S_{i}+S_{j}}$$

where *C_ij_* is the sum of the lesser values for the habitats shared *y* by both cells *i* and *j. S_i_* and *S_j_* are the total number of habitats present in each cell. Bray–Curtis index values were calculated for all pairs of cells in each bioregion using the *vegdist* function in the *vegan* R package (Oksanen et al. 2013), and then averaged per bioregion.

The spatial clustering of habitats measures the degree to which cells of the same habitat type are grouped together in each bioregion. Ecologically, this measure reflects the extent to which a species perceives the habitat as being homogeneous. To quantify habitat clustering we used the Moran’s I index of spatial autocorrelation. Moran’s I ranges from −1 (total spatial decorrelation) to +1 (total autocorrelation). Thus, values close to −1 for a given habitat would indicate a very fragmented habitat across its range, while habitats with values of Moran’s I close to +1 would exhibit high spatial clustering. Moran’s I was calculated using the following formula:

(2) $$I=\frac{N}{W}\frac{\sum \;i\sum^{\;}i\omega ij\left(x_{i}-\bar{x}\right)\left(x_{j}-\bar{x}\right)}{{\sum_{i}}^{\;}{\left(x_{i}-\bar{x}\right)}^{2}}$$

where *N* is the number of cells in the bioregion being considered, indexed by subscripts *i* and *j; x* = 1 if the habitat is present in the corresponding cell and 0 otherwise; $\bar{x}$ is the mean of *x* (i.e. the fraction of cells harbouring that habitat); ω *_ij_* are elements of a matrix of spatial weights with zeros on the diagonal and 1 if cell *i* is a directly adjacent neighbour of cell *j*; and W is the sum of all *ω_ij_* We used the *raster* package in R to calculate Moran’s I for each habitat within each bioregion, and we averaged the values across habitats, obtaining thus a single habitat clustering value per bioregion.

### Statistical analyses

The shapes of NARs and SARs were quantified by fitting power functions to the relationships obtained across the 100 replicates between network properties and area size using nonlinear least squares (NLS) regressions with the *nls* function in R. These power functions allowed us to obtain a single value (the z exponent) representing the rate of increase of each dependent variable versus the predictor (i.e. area).

To assess the effects of habitat clustering and environmental heterogeneity on the shape of SARs and NARs, we tested the relationship between our selected spatial and environmental predictors and the scaling exponents (z) of these relationships using linear regressions. Before performing regressions, we carried out commonality analysis (CA) as a mean of variable selection to avoid multi-collinearity (Seibold and McPhee 1979, Prunier et al. 2015). CA allows for the systematic evaluation of the relative contribution of each predictor variable to the predictive power of a linear regression model (cf. the Supporting information for further details). The selected predictors (i.e. those with low degree of collinearity and good correlation with dependent variables) were used in linear regressions to quantify their relations to SARs and NARs scaling exponents across bioregions. We used generalized additive models to facilitate the visual representation of the raw data. All analyses were performed in R (<www.r-project.org>).

## Results

### Network-area relationships

**Figure 2:**
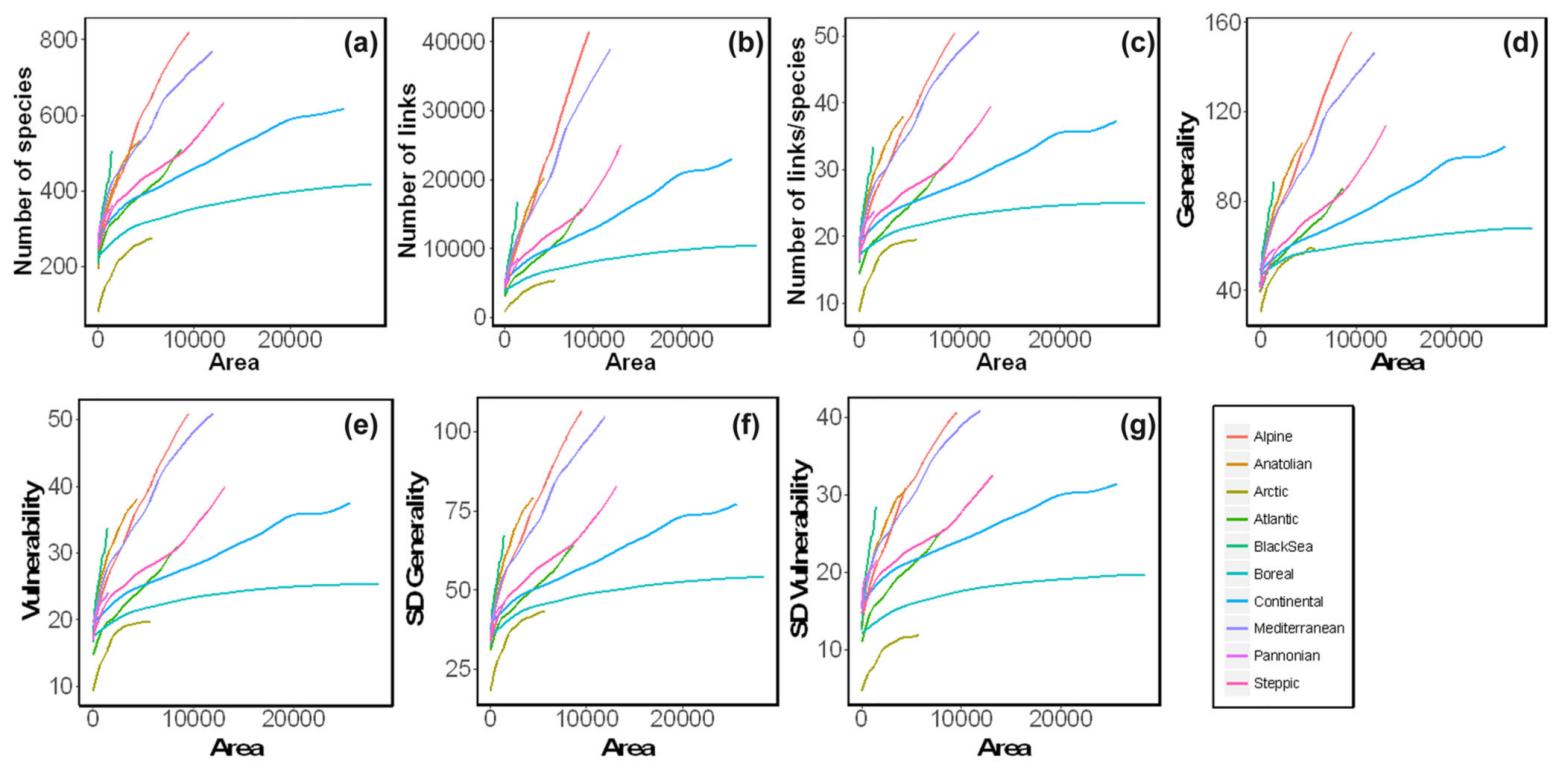
The spatial scaling of network complexity properties across biogeographical regions in Europe. (a) Number of species, (b) links, (c) links per species, (d) mean generality (e) mean vulnerability, (f) standard deviation of generality and (g) standard deviation of vulnerability increase differently with area size across biogeographical regions (colour lines). Yet, total area and maximum values of network properties differ among biogeographical regions, which increases the visual differences between them. For a detailed description of the network properties see Methods. Lines represent a generalized additive model fit to data points. See the Supporting information for figure with data points.

Amongst the different network properties tested here, all complexity measures (i.e. number of species, links, links/species, mean generality, mean vulnerability, SD of generality and SD of vulnerability) increased with area size (Fig. 2), while the spatial scaling (the exponent (*z*) of the power function) differed across bioregions (Supporting information). For instance, the *z*-exponents of the number of species with area ranged from 0.08 (Pannonian) to 0.38 (Alpine). For the other complexity metrics, a general pattern emerged. For most bioregions, while the number of links per species, mean and SD of generality and mean and SD of vulnerability scaled with area at the same rate as the number of species (i.e. very similar *z* of the fitted power functions), the number of links scaled twice as fast (Supporting information). That is, the scaling exponents of the number of links with area ranged between 0.16 (Pannonian) and 0.77 (Alpine), meaning that communities were gaining twice as many links as species with increasing area. This was consistent across bioregions. Exceptions to this pattern were the Arctic and Boreal regions, which showed a scaling in the number of links with area (*z* = 0.46 and *z* = 0.25, respectively) closer to that observed for the number of species (*z* = 0.31 and *z* = 0.15). This in turn slowed down (i.e. reduced z-exponents) the spatial scaling of the other complexity properties (Fig. 2).

**Figure 3:**
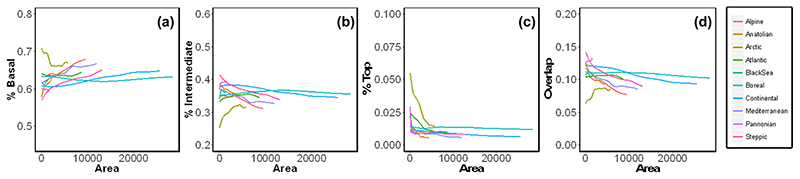
Scale-invariant network properties. Relationship of the percentage of (a) basal, (b) intermediate and (c) top species with area and consumers’ diet overlap across biogeographical regions in Europe. The proportions of species per trophic level showed similar values across spatial scales and across biogeographical regions. Lines represent a generalized additive model fit to data points. Supporting information for figure with data points.

In contrast, the proportion of species per trophic level and the proportion of overlap in the consumers’ diet, were largely scale-invariant (Fig. 3). The proportion of basal, intermediate and top species showed similar values from local to regional spatial scales, including the values for the metaweb (i.e. at the European level without considering bioregions; Fig. 3, Table 2). The Arctic was an exception, showing the largest variation in these proportions across spatial scales (Fig. 3, Table 2).

### Contribution of species richness to NARs

**Figure 4:**
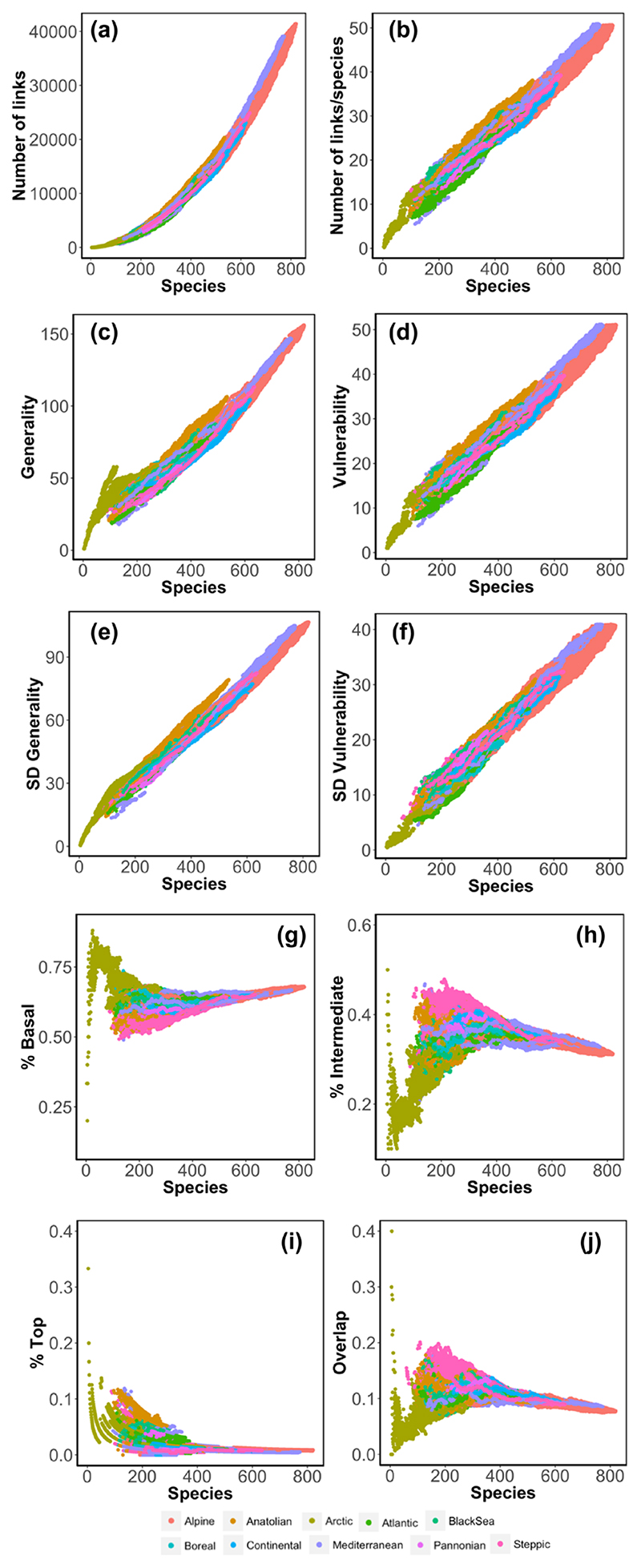
Relationship of network properties with species richness across biogeographical regions in Europe. Network complexity properties, i.e. (a) number of links, (b) links per species, (c) mean generality, (d) mean vulnerability, (e) standard deviation of generality and (f) standard deviation of vulnerability, strongly correlated with species richness in all biogeographical regions. In contrast, vertical diversity properties, i.e. (g) proportion of basal, (h) intermediate, (h) top species and (j) consumers’ diet overlap, do not correlate with species richness. Not every bioregion has the same number of species and, therefore, some are not represented along the whole range of species richness.

To determine the contribution of the spatial scaling of species richness (SARs) to the scaling of the remaining network properties (NARs), we analysed the relationship between each property and species richness for each bioregion (Fig. 4; Supporting information) and we built networks with an equal number of species while changing the structure of the network and compared the patterns observed (Supporting information). The linear regressions showed that all network complexity properties were highly predictable by species richness in all bioregions (Fig. 4a–d), with a mean adjusted-R^2^ = 0.97 ± 0.03 (SD) (Supporting information). Although a relationship between food web complexity measures and species richness was expected, it is important to notice the consistency of slope values across bioregions. For instance, the number of links scaled exponentially with species richness at similar rates across all bioregions (slope = 1.88 ± 0.14 in log–log space; Fig. 4a; Supporting information).

The subsampled networks generated with an equal number of species showed extremely similar network–area relationships than those observed with the original networks when the links between species were taken from the metaweb (null model-1) (Supporting information). That is, most of the ratios between the estimated z-exponents obtained from the power function fits for the null model networks and the estimated z-exponents of original networks were really close to 1 (indicating strong similarities) for all bioregions except from Arctic and Boreal, which showed slightly larger differences (Supporting information). Interestingly, although consumers’ overlap was not influenced by species richness, the values obtained in the subsampled assemblages were considerably larger. The similarity between the patterns observed using the subsampled networks and the original ones, indicated that there is a strong influence of species richness on the spatial scaling of network complexity. This strong influence of species richness was corroborated by the constancy observed of the normalised properties (i.e. network properties divided by the number of species) across spatial scales (Supporting information).

The null model-2 allowed us to determine whether there is any further contribution of space beyond the effect of species richness and the inherited properties of the metaweb. While most properties showed patterns similar to the original networks, the proportion of species per trophic level and the percentage of consumers’ diet overlap showed strong differences (Supporting information). In the original networks, SARs differed across trophic levels, being faster for lower trophic levels in all regions (Supporting information). Yet, the proportion of species per trophic level did not show strong relationships with the number of species for most regions (mean adjusted-R^2^ = 0.39 ± 0.27). The estimated slope of the relationships was close to 0, indicating that the proportion of species per trophic level did not change significantly as total species richness increases (Fig. 4g-i; Supporting information for an explanation on how the proportion of species per trophic level is constant while the species–area relationship differs). Both null models also showed a constant proportion of species per trophic level as species richness increases. Yet, in the random networks all species were considered intermediate since it is very unlikely that a species has no prey or that it is not predated by any other species (Supporting information), which indicates that the observed proportions of species per trophic level in the original networks are inherited from the metaweb. Similarly, in the original networks the proportion of diet overlap among consumers was small and it did not change significantly with species richness (Fig. 4j). Conversely, in the generated networks, consumers’ diet overlap was significantly higher and it increased with spatial scale and species richness, suggesting that in the original networks there might be a spatial structuring of species that minimizes consumers’ diet overlap.

### Environmental drivers of species–area relationships

**Figure 5:**
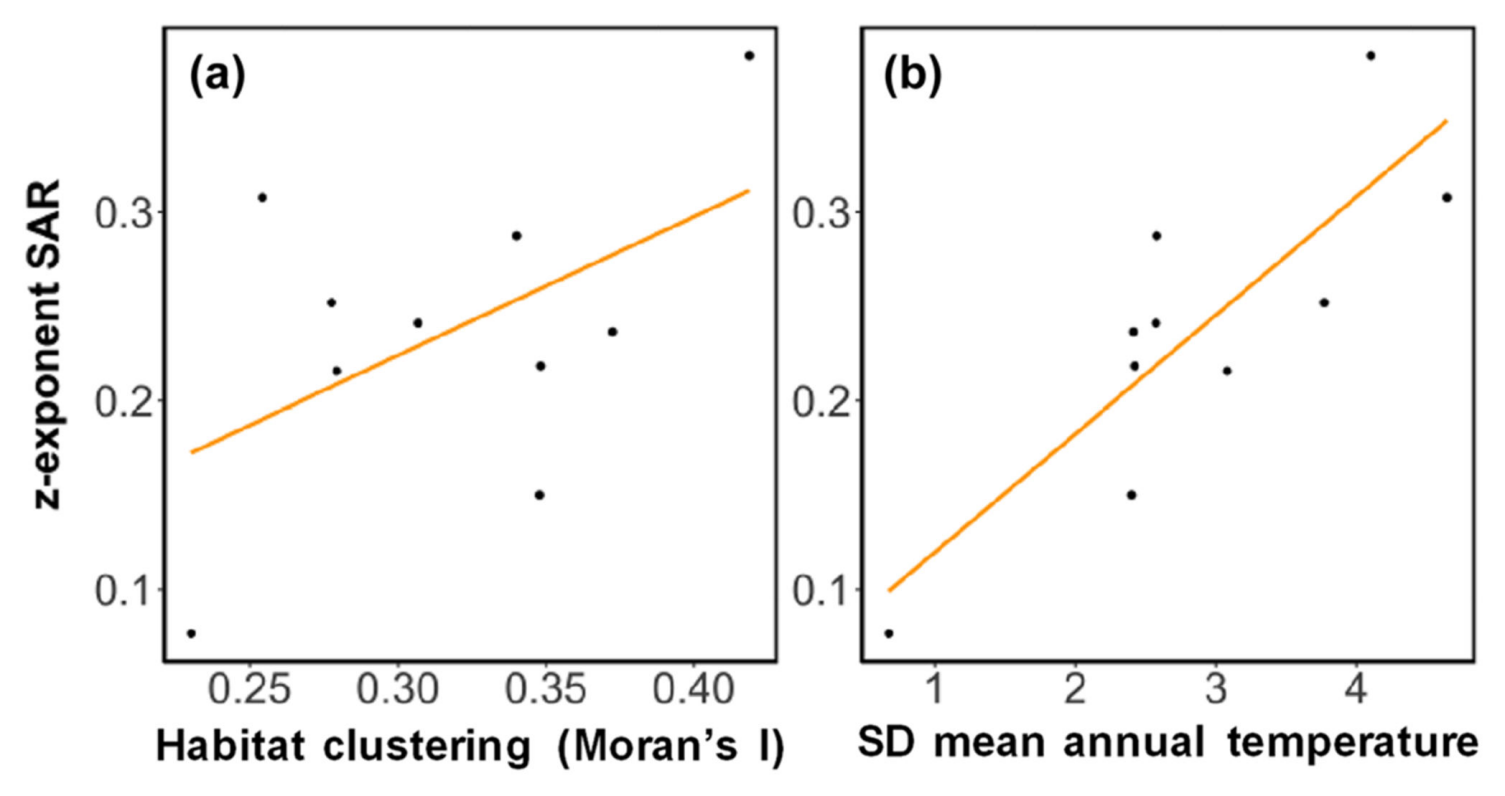
Relationship between habitat heterogeneity and temperature variability, and the scaling of SARs. Linear regression between (a) habitat clustering (quantified as Moran’s I) and (b) spatial variability in the mean annual temperature (quantified as the standard deviation of the mean annual temperature) across European bioregions, and the scaling exponent (z) of species-area relationships within them. Linear regression between response and predictors is given by: *y* = 0.54*x*_1_ + 0.06*x*_2_. R^2^ = 0.83, p < 0.01 on 7 degrees of freedom. Each point represents a bioregion and lines are predictions from linear regression models.

Since differences observed across European bioregions in the spatial scaling of food web structure were primarily driven by the differences in SARs, we investigated the environmental factors determining those latter differences (Supporting information). Analysis of the correlation between predictor variables indicated a high degree of collinearity between them (Supporting information), which would bias the results of classical statistical models (Seibold and McPhee 1979, Prunier et al. 2015). Commonality analysis revealed that, among the predictor variables considered, the standard deviation of the mean annual temperature across cells in each region (i.e. spatial variation), and the spatial clustering of habitats (i.e. Moran’s Index), were the most robust predictors of SARs scaling exponents *z* (Supporting information). Together, the spatial variation in the mean annual temperature and the spatial clustering of habitats within each biogeographical region explained 83.17% of the variability observed in the exponents of SARs (t-statistic = 4.87, p-value < 0.01 and t-statistic = 2.38, p-value < 0.05, respectively on seven degrees of freedom) (Fig. 5). Therefore, regions with larger spatial variability in their mean annual temperature and higher habitat clustering (i.e. more continuous habitat patches) tended to accumulate species faster as area sampled increased, which in turn affected the spatial scaling of network structure.

## Discussion

The spatial scaling of biodiversity has been traditionally understood as exclusively the scaling of species richness with area size (Arrhenius 1921, Rosenzweig 1995, Lawton 1999). However, species interactions are intrinsic components of ecological communities. As such, understanding how the network of interactions changes across spatial scales is pivotal to fully understand how biodiversity changes with area (Galiana et al. 2018). We used a network of potential trophic interactions between European terrestrial vertebrates to analyse the spatial scaling of network structure across biogeographical regions. Although we found marked differences in the spatial scaling of network complexity across bioregions, we also found striking universalities. The proportion of species per trophic level and the proportion of diet overlap among consumers were constant across spatial scales and bioregions. Moreover, all the differences found in the spatial scaling of network complexity were mirrored by differences in the spatial structure of species richness, suggesting that the scaling of species richness is a strong driver of the scaling of network properties.

The effect of species richness on many other aspects of network structure has repeatedly been studied in local communities (Bengtsson 1994, Vermaat et al. 2009, Dunne et al. 2013). The variation in many local food web properties is largely driven by changes in species richness (Bengtsson 1994, Dunne et al. 2013, Eklöf et al. 2013). However, whether these correlations between species richness and network structure hold across large ranges of species richness and across different spatial scales was, so far, unknown. Here, we showed that species richness was enough to explain most of the geographical variability of the spatial scaling of network complexity in terrestrial vertebrate food webs. Network complexity strongly correlated with species richness in extremely similar ways (i.e. same slopes) across all bioregions in Europe. This suggests that patterns previously observed at local scales also hold at large spatial scales, covering a much wider range of species richness (from 5 to 820 species) and across multiple bioregions, where communities are subject to different environmental, historical and evolutionary conditions.

Species richness did not explain, however, the patterns observed for the proportion of species per trophic level or the proportion of diet overlap among consumers. The fraction of species per trophic level was traditionally thought to be constant among networks across a wide range of species richness, displaying a pyramidal shape where species richness consistently decreased with trophic level (Cohen et al. 1990, Pimm et al. 1991). However, further research found that although trophic diversity structure is generally pyramidal (Turney and Buddle 2016), the distribution of species richness per trophic level can also depend on external factors such as latitude, net primary productivity or ecosystem type (Vermaat et al. 2009, Turney and Buddle 2016), and that it might depend on the total number of species in the community (Martinez and Lawton 1995, Vermaat et al. 2009, Turney and Buddle 2016) and the spatial scale considered (Martinez and Lawton 1995, Wood et al. 2015). The proportions of species per trophic level in our terrestrial vertebrate food webs were constant across bioregions, spatial scales and species richness. The comparisons with our null models indicated that the observed proportions were inherited from the metaweb. These proportions decreased from basal to top species, generating pyramidal food webs. It is important to notice, however, that, in this study basal species correspond to vertebrate species feeding on resources such as invertebrates, plants and carrion, instead of primary producers themselves, which are usually considered the basal species in food web studies. Although our definition of basal species differs from the classical basal species concept in food webs, given that we use the same definition across all regions and spatial scales the universality found should prevail. Similarly, the proportion of top consumers in our communities was very low due to the potential nature of our metaweb. That is, given that the metaweb is composed by all potential links between species, it is difficult to find a species having no potential predators. Therefore, the proportion of top species might be reduced by the potential nature of the links of the metaweb, while the proportion of intermediate species might be enlarged.

Given the influence of SARs on the spatial scaling of the network properties of our European terrestrial communities, understanding the factors and mechanisms promoting variability in SARs was sufficient to examine the potential causes of the changes in food web structure with the area. Multiple mechanisms have been proposed to explain the shape of SARs and, in particular, the scaling exponent *z* (Drakare et al. 2006, Triantis et al. 2012). Although at the European scale, we recovered a multiphasic SAR with clear transitions between bioregions (Supporting information), indicating the potential crossing of different evolutionary provinces where assemblages do not share evolutionary history and dispersal is strongly limited (Rosenzweig 1995, Drakare et al. 2006, Triantis et al. 2012), we focused our analyses at the bioregions scale, where the power function provided a good fit for all regions (Supporting information).

Different exponents of SARs across bioregions indicate that these relationships vary across environmental conditions. The spatial variability of mean annual temperature and the spatial clustering of habitats were the main correlates of the spatial scaling of diversity across Europe. The spatial variability in temperature is one aspect of the environmental heterogeneity present in each biogeographical region. This agrees with niche differentiation theory: the larger the range of environmental conditions, the larger the regional coexistence due to niche differentiation and adaptation, which in turn promote a faster accumulation of species with area (Chesson 2000, Amarasekare 2003). Similarly, habitat heterogeneity has been traditionally identified as an important underlying component of the scaling exponent of SAR. The larger the area sampled, the larger the number of different habitats encountered sustaining a larger set of species (Boecklen 1986, Drakare et al. 2006, Shen et al. 2009). Yet, the role of the spatial clustering of habitats on the spatial scaling of diversity was, so far, seldom explored (Shen et al. 2009, Rybicki and Hanski 2013). Our analyses indicate that more continuous habitat patches facilitate a faster accumulation of species with area, stressing the potential effects of habitat fragmentation on the spatial scaling of biodiversity.

While in our study, these mechanisms affected the spatial scaling of network complexity only indirectly through the effect on the spatial scaling of species richness, environmental factors can directly affect network structure across spatial scales. Habitat structure has been shown to have direct effects on biotic interactions. For example, habitat loss or modification can alter biotic interactions (Tylianakis et al. 2007, Fortuna et al. 2013) and ecosystem functioning (Gonzalez et al. 2020) and stability (Morris 2010, Gonzalez et al. 2011, McWilliams et al. 2019), without large variations in species richness. This highlights the need for incorporating information on the spatial scaling of network structure to fully assess the impacts of habitat modification on biodiversity and ecosystem functioning (Gonzalez et al. 2011, 2020, Cardinale et al. 2012). Moreover, landscape heterogeneity increases species sorting (i.e. different habitat preferences between species), which can generate modules in the network and promote a modular structure (Pimm and Lawton 1980, Araujo et al. 2018). Our food webs, however, presented extremely low values of modularity. This is most likely due to the way in which networks were built, which encapsulate all potential interactions between species and, therefore, might prevent the existence of modules.

One potential explanation for the large explanatory power of species richness for the network patterns observed could be the fact that interactions between species were fixed. That is, every time a pair of interacting species of the metaweb co-occurred in space, we assumed they interact. However, some interactions can be context dependent (Poisot et al. 2012, Chamberlain et al. 2014): even if two species co-occur in space, they may not interact (or do it very weakly) if, for instance, the environment is not favourable enough (Poisot et al. 2011, 2012). Therefore, the use of fixed and qualitative (i.e. unweighted) interactions might limit our understanding of the spatial scaling of network structure given that further variation in the intensity of species interactions, and in turn in network structure, might occur in nature. To what extent interaction context-dependency is anecdotal or a general phenomenon requires detailed information not possible to obtain in our data. Future analyses of spatial scaling of species and interactions, where ideally information on interaction strength is accessible, are needed to deepen our knowledge on how ecological networks change across spatial scales, what is the influence of species richness on the scaling and which are the main environmental drivers. Here, we have revealed which network properties are scale-dependent and strongly influenced by the number of species, and which are scale and geographically invariant, indicating that ecological communities might be spatially structured to be able to preserve fundamental properties, such as a low consumers’ diet overlap.

## Supplementary Material

Supporting Information

## Figures and Tables

**Table 1:** Metaweb properties. Network complexity metrics: number of species, links, links per species, connectance, mean indegree and mean outdegree. Vertical diversity metrics: proportion of basal, intermediate and top species. Network modularity indicates the presence of densely linked groups within the network.

Property	Value
Number of species	1140
Connectance	0.056
Number of links	67 201
Links/species	61.09
Generality	211.32
Vulnerability	61.43
SD generality	144.89
SD vulnerability	50.53
Proportion of basal	0.7
Proportion of intermediate	0.28
Proportion of top	0.02
Consumer’s overlap	0.72
Modularity	0.21

**Table 2:** Proportion of species in each trophic level at local and regional spatial scales across the biogeographical regions in Europe. Local scale corresponds to the average proportion of species in each trophic level across all 10-km^2^ cells from each bioregion. Regional scale corresponds to the network resulting from the aggregation of all the cells for each biogeographical region. The Metaweb corresponds to all European bioregions grouped.

	% Basal		% Intermediate		% Top	
	Local	Regional	Local	Regional	Local	Regional
Metaweb	0.63 ± 0.06	0.71	0.33 ± 0.06	0.28	0.04 ± 0.03	0.05
Alpine	0.63 ± 0.03	0.68	0.34 ± 0.04	0.31	0.02 ± 0.01	0.008
Arctic	0.71 ± 0.07	0.67	0.21 ± 0.07	0.31	0.07 ± 0.04	0.014
Atlantic	0.64 ± 0.02	0.65	0.32 ± 0.03	0.34	0.03 ± 0.02	0.001
BlackSea	0.61 ± 0.03	0.63	0.34 ± 0.04	0.36	0.03 ± 0.02	0.01
Boreal	0.64 ± 0.03	0.63	0.35 ± 0.03	0.36	0.02 ± 0.01	0.01
Continental	0.62 ± 0.02	0.65	0.36 ± 0.03	0.35	0.02 ± 0.01	0.006
Mediterranean	0.61 ± 0.04	0.68	0.36 ± 0.04	0.32	0.02 ± 0.02	0.005
Pannonian	0.61 ± 0.02	0.59	0.36 ± 0.02	0.39	0.03 ± 0.01	0.01
Steppic	0.56 ± 0.03	0.65	0.41 ± 0.04	0.34	0.02 ± 0.02	0.007
Anatolian	0.56 ± 0.03	0.64	0.35 ± 0.05	0.31	0.02 ± 0.01	0.008
