# Supplementary material for: The spatial scaling of food web structure across European biogeographical regions
Source: Ecography. Author manuscript; Available in PMC 2023 Jan 6. (PMC7614028; doi:10.1111/ecog.05229)
Supplement: Supporting Information [file EMS130718-supplement-Supporting_Information.docx]

## SUPPLEMENTARY MATERIAL OF THE SPATIAL SCALING OF FOOD WEB STRUCTURE ACROSS EUROPEAN BIOGEOGRAPHICAL REGIONS

**Content**

- **Appendix S1.** Network structural properties used to quantify the structure of simulated food webs.
- **Appendix S2.** Statistical analyses for the determinants of the Species-Area relationship.
- **Appendix S3.** Power function fit for all network properties analysed.
- **Appendix S4.** Relationships of network properties with species richness.
- **Appendix S5.** Diﬀerent increase in species richness across trophic levels.
- **Appendix S6.** Species-Area relationship across biogeographical regions in Europe.
- **Appendix S7.** Contribution of each biogeographical region to the global species-area relationship in Europe.
- **Appendix S8.** Network-Area relationship across biogeographical regions in Europe showing data points.
- **Appendix S9.** Null models results to test the effects of species richness on the spatial scaling of network structure.

**Appendix S1*.*** **Network structural properties used in this study to quantify the structure of simulated food webs.** The name of the properties, their abbreviations and a brief description are presented.

| **Property** | **Abbrev.** | **Description** |
| --- | --- | --- |
| Species richness | S | Number of species in the community. |
| Number of links | L | Total number of trophic interactions in the food web. |
| Links per species | L/S | Average number of links (i.e. interactions) per species. |
| Connectance | C | Fraction of realised links out of all the possible links in the food web. Calculated as: C = L/S2 |
| Generality | Gen | Mean number of resources per consumer. |
| Vulnerability | Vul | Mean number of predators per resource. |
| Standard deviation of generality | GenSD | SD of number of resources per consumer. |
| Standard deviation of vulnerability | VulSD | SD of number of predators per resource. |
| Fraction of basal species | B | Fraction of basal species, out of the total number of species, in the food web. Basal species are those with no prey (i.e. no incoming links). |
| Fraction of intermediate species | I | Fraction of species in the food web having both prey and predators (i.e. sitting in the ‘middle’ of the food web). |
| Fraction of top species | T | Fraction of top consumers in the food web (i.e. species that do not have predators). |
| Consumer Overlap | Overlap | A measure the quantifies the fraction of consumers sharing at least one prey. This is calculated as the connectivity = L / ((Pred * (Pred-1)/2)) of the networks of shared resources between consumers, where a link is drawn between to consumers when they share at least one prey. |

Additionally, network modularity (M) was quantified using the modularity function (*Q*) proposed by Newman and Girman (Newman & Girvan, 2004):

$$Q= \frac{1}{2m} \sum_{i,j} \left( A_{ij}- P_{ij} \right) \delta(g_{i}, g_{j})$$

where *m* is the number of edges in the network, *A_ij_* are the adjacency matrix elements (1 if a link between vertices *i* and *j* exists and 0 otherwise), *P_ij_* is the expected number of links between *i* and *j* (i.e., *k_i_k_j_/2m*, with *k* the degree of a vertex), *g_i_* is the compartment to which vertex *i* belongs (as described in (Barber, 2007). $\delta(r,s)$ = 1 if *r* = *s* and 0 otherwise. We used the random-walk based algorithm walktrap (Pons & Latapy, 2006) implemented in the R package *igraph* (Csardi & Nepusz, 2006)*.* Yet, the values of modularity obtained ranged from 0 to 0.07 across all bioregions, indicating that the networks analysed showed extremely low modularity values. Therefore, we excluded modularity from the analyses.

**Appendix S2.** Statistical analyses for the determinants of the Species-Area relationships.

We hypothesised that the variation across SARs scaling in different European bioregions was explained by a set of environmental and habitat complexity variables (see methods). We then set off to assess the relationship between these predictor variables and the z-exponent of SARs across bioregions. A single SAR was fitted to each bioregion considering all data points from the 100 replicates per bioregion. However, the high correlation detected among many of the variables (Fig. S1) suggested a potentially strong multi-collinearity eﬀect on the explanatory power of these variables. Predictor variables considered included: total area of the bioregion (Total Area), Bray-Curtis dissimilarity between habitat composition across cells of the gridded map of the bioregion (Bray-Curtis), Moran’s I spatial autocorrelation index across habitats within each bioregion, number of habitats (No. of habitats), average of the mean annual temperature and precipitation across cells in the bioregion (Mean Temp. and Mean Prec. respectively), the variability (measured as the standard deviation) in the mean annual temperature and precipitation across cells in the bioregion (SD Mean Temp. and SD Mean Prec., respectively), temperature and precipitation seasonality during the year averaged across map cells (Mean Seas. Temp. and Mean Seas. Prec., respectively), and the variability (i.e. SD) in temperature and precipitation seasonality across cells (SD Seas. Temp. and SD Seas. Prec., respectively).

**Figure S1. Correlation among predictor variables for the scaling of SARs**. Scatterplots of the pairwise relationships between variables and their distributions are shown. Numbers on the upper triangle of the matrix are Pearson’s correlation coeﬃcients and stars indicate their significance based on the p-value (· < 0.05, * < 0.01, ** < 0.001). Red lines are fitted linear regressions to the data. Names of the variables are explained in the text.

Because of the potential eﬀects of multi-collinearity, we used a Commonality Analysis (CA) (Prunier et al. 2015) to find the best predictors for the scaling exponent (z) of SARs. In brief, CA is a statistical technique that oﬀers information of the statistical relationship between several predictor variables when they are used to explain a dependent (i.e. response) variable using multivariate linear regression. In our case the response variable is the *z* exponent of SARs for each bioregion. By considering correlations among predictors, CA calculates variance inflation factors (VIF). Additionally, CA provides information on the fraction of the model fit that is due to suppression among predictor variables, their unique vs. common contributions to the model explanatory abilities, and the mismatch between the variables’ coefficient when used alone vs. when considered jointly in the linear regression. All this information can be used to select the variables that explain best the model fit, by removing those that artificially inflate the model’s fit. Here we provide an example of how CA was used to remove variables having a potential artificial eﬀect on the model’s capacity to explain variability in the data. Because we had more predictor variables than data points in our dataset, we split the set of predictor variables into two subsets and carried out CA independently on both sets. We then carried out a joint CA on the resulting selected variables from each independent CA to ensure these were still a good fit to the observed data without artificially inflating the model’s fit. Performing CA over each subset of predictor variables (vs. the same response variable, *z*) to results in the values shown in Tables S1 and S2.

**Table S2. Results of CA performed over the subset of predictor variables including: Bray-Curtis, Moran’s I. No. of Habitats, Mean Temp. and SD Mean Temp..** This table shows the linear regression coeﬃcients both jointly and in isolation (rs and betas, respectively), the standard error of the mean (StdErr), the lower and upper limits of the confidence intervals (CIinf, CIsup), p-values of significance based on 200 bootstrapping iterations, total contribution to the model (split into unique and common) and the variance inflation factor (VIF) for each predictor variable considered in the model.

| **Variable** | **rs** | **betas** | **StdErr** | **CIinf** | **CIsup** | **p-value** | **Unique** | **Common** | **Total** | **VIF** |
| --- | --- | --- | --- | --- | --- | --- | --- | --- | --- | --- |
| Bray-Curtis | 0.757 | -0.049 | 0.291 | -1.364 | 6.818 | 0.874 | 0.0007 | 0.515 | 0.515 | 3.353 |
| Moran’s I | 0.539 | -0.036 | 0.381 | -4.119 | 7.364 | 0.929 | 0.0002 | 0.261 | 0.261 | 5.739 |
| No. of Habitats | 0.724 | 0.525 | 0.451 | -4.217 | 3.153 | 0.308 | 0.034 | 0.437 | 0.472 | 8.052 |
| Mean Temp. | -0.346 | 0.410 | 0.254 | -2.913 | 1.241 | 0.182 | 0.066 | 0.042 | 0.108 | 2.547 |
| SD Mean Temp. | 0.880 | 0.871 | 0.242 | -3.156 | 6.311 | 0.023 | 0.327 | 0.368 | 0.696 | 2.314 |

**Table S3. Results of CA performed over the subset of predictor variables including: Mean Seas. Temp., SD Seas. Temp., Mean Prec., SD Mean Prec., Mean Seas. Prec., and SD Seas. Prec.** This table shows the linear regression coeﬃcients, both jointly and in isolation (rs and betas, respectively), the standard error of the mean (StdErr), the lower and upper limits of the confidence intervals (CIinf, CIsup), p-values of significance based on 200 bootstrapping iterations, total contribution to the model (split into unique and common) and the variance inflation factor (VIF) for each predictor variable considered in the model.

| **Variable** | **rs** | **betas** | **StdErr** | **CIinf** | **CIsup** | **p-value** | **Unique** | **Common** | **Total** | **VIF** |
| --- | --- | --- | --- | --- | --- | --- | --- | --- | --- | --- |
| Mean Seas. Temp. | -0.065 | 0.420 | 0.326 | -10.725 | 1.786 | 0.288 | 0.028 | -0.024 | 0.004 | 6.325 |
| SD Seas. Temp. | 0.638 | 0.527 | 0.196 | -0.479 | 5.225 | 0.075 | 0.121 | 0.265 | 0.386 | 2.288 |
| Mean Prec. | 0.156 | 0.049 | 0.325 | -14.453 | 1.720 | 0.890 | 0.0004 | 0.023 | 0.023 | 6.271 |
| SD Mean Prec. | 0.525 | 0.751 | 0.333 | -0.964 | 11.321 | 0.110 | 0.085 | 0.177 | 0.262 | 6.616 |
| Mean Seas. Prec. | 0.202 | 0.278 | 0.261 | -0.922 | 4.712 | 0.364 | 0.019 | 0.020 | 0.039 | 4.054 |
| SD Seas. Prec. | 0.534 | 0.389 | 0.258 | -7.534 | 1.718 | 0.229 | 0.038 | 0.233 | 0.271 | 3.961 |

Model fit resulting from the CA over the first subset of predictor variables (Table S1) was high (89.9% of the variance explained). However, the VIFs (Table S1) and the percentage of suppression in the model fit (19.98%) detected were both high. This suggests that at least some of the variables have an artificial (i.e. indirect) eﬀect on model fit, and hence its explanatory capabilities. From the values shown in Table S1, we can see that the total contribution to model fit of mean temperature is very low (0.108), so this is a good candidate to remove from the analysis. Also, the VIF for the number of habitats is extremely high (8.052), making this variable also a good candidate to be removed.

We removed predictor variables from our first subset of predictors following the classes of criteria outlined above, in terms of VIF values, amount of total contribution, and comparisons of common vs. unique contributions, until the suppression of model fit due to multi-collinearity was reduced to 0%. Even though there are no hard guidelines on how specific values to apply to each criteria, commonly used values include: VIF < 2, total contribution > 5 % (i.e. 0.05), and unique contributions >> common contributions. After applying these criteria to both subsets of predictors independently, we ended up with two main predictors that were able to explain 83.17% of the model variability: the variability of the mean annual temperature across the spatial extent of the bioregion (SD Mean Temp.) and the Moran’s I index of habitats spatial autocorrelation (Moran’s I). Linear regression of these two predictors on the SARs scaling exponent (z ~ SD Mean Temp. + Moran’s I) showed that there is a significant relationship between predictors and dependent variables (adjusted R^2^ = 0.7836, p-value < 0.01, respectively).

After following the same heuristics for the CA on the second subset of predictor variables (Table S2) as above, we selected SD Seas. Temp., SD Prec., and SD Seas. Prec., as the variables that produced the best linear regression fit to the response variable (*z*) from the second subset of variables. Linear regression on SARs scaling exponents showed a significant relationship between these predictors and the dependent variable (adjusted R^2^ = 0.8547, p-value < 0.01).

To confirm that the final set of predictor variables selected, including both those derived from the CA over the first set of predictors and those from the CA over the second set (Tables S1 and S2), we conducted CA again over the selected variables, namely: Moran’s I, SD Mean Temp., SD Seas. Temp., SD Prec., and SD Seas. Prec. This revealed a model suppression of 32.19%, most likely due to the high correlation between two of the variables included in the model: SD Mean Temp. and SD Seas. Temp. (Pearson’s correlation = 0.65). Since both of these variables quantify, to some extent or another, the variability in temperature throughout the year, it makes sense that they are correlated. This suggests that removing either of them would not have impacted our interpretation of the results. We thus decided to remove SD Seas. Temp. from the model because keeping the variability in the mean temperature as one of our predictor variables was deemed more intuitive in terms of explaining our results on ecological grounds. After removing SD Seas. Temp. from the model, the suppression fraction dropped to 0%, while keeping a high model fit score: 94.09%.

In spite of this, the unique contribution, when compared to the common contribution to the model fit, is only really significant for the predictor variables selected from the first subset: Moran’s I and SD Mean Temp.. Moreover, the linear regression incorporating the 4 remaining variables, even though yielding a better fraction of the variability observed in the data that is explained by the model (adjusted R^2^ = 0.8122 vs. 0.7836 from the model incorporating Moran’s I and SD Mean Temp. only), is only statistically significant for SD Mean Temp (Table S4). This suggests that the simplest model, incorporating only two variables is a better fit to the data. This observation is corroborated by Akaike Information Criteria scores for both models: -32.84 for the 4 predictors model vs. -32.06 for the model including Moran’s I and SD Mean Temp. (Table S4), which by virtue of their similarity suggest that choosing the model with the less number of variables is appropriate. Thus, both lines of evidence (i.e. better statistical significance of predictors, and AIC scores) suggest that Moran’s I and SD Mean Temp. are the best predictors of the scaling of NARs.

**Table S4. Comparison between the last 2 candidate models relating environmental variables to SAR z-exponent after variable selection via Commonality Analysis.** Significance (i.e. p-value) of predictor variables is indicated by symbols: * < 0.05, ** < 0.01.

| **Model** | **Predictor variables and significance** | **p-value** | **Adjusted R^2^** | **AIC** | **Model selected** |
| --- | --- | --- | --- | --- | --- |
| 1 | Moran’s I (*)  SD Mean Temp. (**) | 0.002 | 0.7836 | -32.06 | ✓ |
| 2 | Moran’s I  SD Mean Temp. (**)  SD Prec.  SD Seas. Prec | 0.011 | 0.8122 | -32.84 |  |

Thus, we can conclude that annual temperature variability and habitat clustering (i.e., spatial autocorrelation) within bioregions are good predictors of the scaling of species number with area. Regions with more variability in temperature and higher habitat clustering tend to accumulate species faster as area sampled increases.

**Appendix S3**. Power function fit for all network properties analysed using the 100 replications for each biogeographical region. Fit of each network property of each biogeographical region to a power function (f(x) = cx^z^), where c is the scaling factor and z is the exponent parameter that determines the function’s rates of growth and its overall shape. We used nonlinear least squares (NLS) with the ’nls’ function in R.

| Parameters | Region | Property | Estimate | Std. Error | t-value |
| --- | --- | --- | --- | --- | --- |
| c | Alpine | species | 24.08 | 0.07 | 368.28 |
| z | Alpine | species | 0.38 | 0.00 | 1215.59 |
| c | Alpine | links | 36.28 | 0.21 | 169.19 |
| z | Alpine | links | 0.77 | 0.00 | 1137.03 |
| c | Alpine | links per sp | 2.48 | 0.01 | 380.35 |
| z | Alpine | links per sp | 0.32 | 0.00 | 1054.93 |
| c | Alpine | generality | 4.01 | 0.01 | 321.15 |
| z | Alpine | generality | 0.39 | 0.00 | 1088.58 |
| c | Alpine | vulnerability | 2.56 | 0.01 | 381.82 |
| z | Alpine | vulnerability | 0.32 | 0.00 | 1051.85 |
| c | Alpine | SD generality | 4.87 | 0.01 | 493.06 |
| z | Alpine | SD generality | 0.33 | 0.00 | 1402.28 |
| c | Alpine | SD vulnerability | 1.89 | 0.00 | 407.54 |
| z | Alpine | SD vulnerability | 0.33 | 0.00 | 1162.45 |
| c | Alpine | overlap | 0.16 | 0.00 | 1325.43 |
| z | Alpine | overlap | -0.07 | 0.00 | -762.69 |
| c | Alpine | basal | 0.56 | 0.00 | 4427.54 |
| z | Alpine | basal | 0.02 | 0.00 | 687.76 |
| c | Alpine | Top | 0.02 | 0.00 | 637.28 |
| z | Alpine | Top | -0.11 | 0.00 | -574.14 |
| c | Alpine | intermediate | 0.42 | 0.00 | 2450.86 |
| z | Alpine | intermediate | -0.03 | 0.00 | -561.35 |
| c | Anatolian | species | 64.82 | 0.08 | 805.00 |
| z | Anatolian | species | 0.25 | 0.00 | 1572.03 |
| c | Anatolian | links | 355.86 | 0.89 | 400.39 |
| z | Anatolian | links | 0.48 | 0.00 | 1526.19 |
| c | Anatolian | links per sp | 6.25 | 0.01 | 953.13 |
| z | Anatolian | links per sp | 0.21 | 0.00 | 1574.16 |
| c | Anatolian | generality | 11.84 | 0.02 | 603.67 |
| z | Anatolian | generality | 0.26 | 0.00 | 1216.00 |
| c | Anatolian | vulnerability | 6.81 | 0.01 | 938.04 |
| z | Anatolian | vulnerability | 0.20 | 0.00 | 1477.01 |
| c | Anatolian | SD generality | 11.15 | 0.01 | 1044.29 |
| z | Anatolian | SD generality | 0.23 | 0.00 | 1880.49 |
| c | Anatolian | SD vulnerability | 4.40 | 0.00 | 975.14 |
| z | Anatolian | SD vulnerability | 0.23 | 0.00 | 1740.11 |
| c | Anatolian | overlap | 0.18 | 0.00 | 1021.16 |
| z | Anatolian | overlap | -0.07 | 0.00 | -526.31 |
| c | Anatolian | basal | 0.51 | 0.00 | 2805.65 |
| z | Anatolian | basal | 0.03 | 0.00 | 568.58 |
| c | Anatolian | Top | 0.09 | 0.00 | 352.59 |
| z | Anatolian | Top | -0.32 | 0.00 | -711.85 |
| c | Anatolian | intermediate | 0.42 | 0.00 | 1978.60 |
| z | Anatolian | intermediate | -0.02 | 0.00 | -262.84 |
| c | Arctic | species | 19.79 | 0.06 | 353.62 |
| z | Arctic | species | 0.31 | 0.00 | 876.44 |
| c | Arctic | links | 109.25 | 0.47 | 230.24 |
| z | Arctic | links | 0.46 | 0.00 | 854.74 |
| c | Arctic | links per sp | 3.56 | 0.01 | 376.94 |
| z | Arctic | links per sp | 0.20 | 0.00 | 604.50 |
| c | Arctic | generality | 14.61 | 0.02 | 612.75 |
| z | Arctic | generality | 0.16 | 0.00 | 789.82 |
| c | Arctic | vulnerability | 4.03 | 0.01 | 382.12 |
| z | Arctic | vulnerability | 0.19 | 0.00 | 572.92 |
| c | Arctic | SD generality | 6.98 | 0.01 | 519.16 |
| z | Arctic | SD generality | 0.21 | 0.00 | 886.39 |
| c | Arctic | SD vulnerability | 1.46 | 0.00 | 369.78 |
| z | Arctic | SD vulnerability | 0.25 | 0.00 | 729.52 |
| c | Arctic | overlap | 0.04 | 0.00 | 468.63 |
| z | Arctic | overlap | 0.09 | 0.00 | 322.08 |
| c | Arctic | basal | 0.81 | 0.00 | 1979.07 |
| z | Arctic | basal | -0.02 | 0.00 | -362.09 |
| c | Arctic | Top | 0.15 | 0.00 | 391.87 |
| z | Arctic | Top | -0.23 | 0.00 | -644.84 |
| c | Arctic | intermediate | 0.13 | 0.00 | 653.29 |
| z | Arctic | intermediate | 0.10 | 0.00 | 534.21 |

| c | Atlantic | species | 64.51 | 0.09 | 687.11 |
| --- | --- | --- | --- | --- | --- |
| z | Atlantic | species | 0.22 | 0.00 | 1259.51 |
| c | Atlantic | links | 154.14 | 0.61 | 253.75 |
| z | Atlantic | links | 0.50 | 0.00 | 1082.36 |
| c | Atlantic | links per sp | 4.14 | 0.01 | 531.01 |
| z | Atlantic | links per sp | 0.21 | 0.00 | 950.50 |
| c | Atlantic | generality | 11.34 | 0.02 | 545.72 |
| z | Atlantic | generality | 0.21 | 0.00 | 975.75 |
| c | Atlantic | vulnerability | 4.34 | 0.01 | 539.62 |
| z | Atlantic | vulnerability | 0.21 | 0.00 | 945.91 |
| c | Atlantic | SD generality | 10.69 | 0.01 | 806.04 |
| z | Atlantic | SD generality | 0.19 | 0.00 | 1271.61 |
| c | Atlantic | SD vulnerability | 2.96 | 0.01 | 524.45 |
| z | Atlantic | SD vulnerability | 0.23 | 0.00 | 1024.90 |
| c | Atlantic | overlap | 0.10 | 0.00 | 2108.18 |
| z | Atlantic | overlap | 0.00 | 0.00 | 18.27 |
| c | Atlantic | basal | 0.64 | 0.00 | 8402.45 |
| z | Atlantic | basal | 0.00 | 0.00 | -13.55 |
| c | Atlantic | Top | 0.07 | 0.00 | 402.05 |
| z | Atlantic | Top | -0.20 | 0.00 | -612.89 |
| c | Atlantic | intermediate | 0.32 | 0.00 | 3675.40 |
| z | Atlantic | intermediate | 0.01 | 0.00 | 382.48 |
| c | BlackSea | species | 95.56 | 0.35 | 276.80 |
| z | BlackSea | species | 0.22 | 0.00 | 392.89 |
| c | BlackSea | links | 602.10 | 4.20 | 143.34 |
| z | BlackSea | links | 0.44 | 0.00 | 425.05 |
| c | BlackSea | links per sp | 8.12 | 0.02 | 416.42 |
| z | BlackSea | links per sp | 0.19 | 0.00 | 506.50 |
| c | BlackSea | generality | 20.45 | 0.05 | 402.14 |
| z | BlackSea | generality | 0.19 | 0.00 | 506.77 |
| c | BlackSea | vulnerability | 8.62 | 0.02 | 427.01 |
| z | BlackSea | vulnerability | 0.18 | 0.00 | 502.09 |
| z | BlackSea | SD generality | 16.47 | 0.03 | 493.20 |
| c | BlackSea | SD generality | 0.18 | 0.00 | 595.59 |
| z | BlackSea | SD vulnerability | 5.80 | 0.01 | 419.80 |
| c | BlackSea | SD vulnerability | 0.21 | 0.00 | 577.68 |
| c | BlackSea | overlap | 0.11 | 0.00 | 1169.35 |
| z | BlackSea | overlap | -0.01 | 0.00 | -77.12 |
| c | BlackSea | basal | 0.60 | 0.00 | 3664.89 |
| z | BlackSea | basal | 0.00 | 0.00 | 66.90 |
| c | BlackSea | Top | 0.05 | 0.00 | 216.07 |
| z | BlackSea | Top | -0.21 | 0.00 | -266.87 |
| c | BlackSea | intermediate | 0.34 | 0.00 | 1735.54 |
| z | BlackSea | intermediate | 0.01 | 0.00 | 116.17 |
| c | Boreal | species | 89.41 | 0.03 | 2636.06 |
| z | Boreal | species | 0.15 | 0.00 | 3765.94 |
| c | Boreal | links | 845.54 | 0.48 | 1759.65 |
| z | Boreal | links | 0.25 | 0.00 | 4166.94 |
| c | Boreal | links per sp | 9.91 | 0.00 | 4607.56 |
| z | Boreal | links per sp | 0.09 | 0.00 | 3975.52 |
| c | Boreal | generality | 27.15 | 0.01 | 4069.75 |
| z | Boreal | generality | 0.09 | 0.00 | 3408.63 |
| c | Boreal | vulnerability | 10.09 | 0.00 | 4600.91 |
| z | Boreal | vulnerability | 0.09 | 0.00 | 3947.83 |
| c | Boreal | SD generality | 19.18 | 0.00 | 5065.92 |
| z | Boreal | SD generality | 0.10 | 0.00 | 4857.31 |
| c | Boreal | SD vulnerability | 5.83 | 0.00 | 2239.29 |
| z | Boreal | SD vulnerability | 0.12 | 0.00 | 2532.98 |
| c | Boreal | overlap | 0.13 | 0.00 | 3054.78 |
| z | Boreal | overlap | -0.02 | 0.00 | -480.89 |
| c | Boreal | basal | 0.63 | 0.00 | 8695.18 |
| z | Boreal | basal | 0.00 | 0.00 | -114.63 |
| c | Boreal | Top | 0.02 | 0.00 | 1914.73 |
| z | Boreal | Top | -0.03 | 0.00 | -483.30 |
| c | Boreal | intermediate | 0.35 | 0.00 | 5164.55 |
| z | Boreal | intermediate | 0.00 | 0.00 | 175.30 |
| c | Continental | species | 54.70 | 0.05 | 1088.57 |
| z | Continental | species | 0.24 | 0.00 | 2444.40 |
| c | Continental | links | 147.24 | 0.34 | 434.07 |
| z | Continental | links | 0.50 | 0.00 | 2076.06 |
| c | Continental | links per sp | 4.79 | 0.00 | 961.13 |
| z | Continental | links per sp | 0.20 | 0.00 | 1797.57 |
| c | Continental | generality | 8.18 | 0.01 | 777.01 |
| z | Continental | generality | 0.25 | 0.00 | 1818.62 |
| c | Continental | vulnerability | 4.92 | 0.01 | 969.81 |

| z | Continental | vulnerability | 0.20 | 0.00 | 1793.80 |
| --- | --- | --- | --- | --- | --- |
| c | Continental | SD generality | 9.26 | 0.01 | 1295.71 |
| z | Continental | SD generality | 0.20 | 0.00 | 2510.60 |
| c | Continental | SD vulnerability | 4.29 | 0.00 | 1179.72 |
| z | Continental | SD vulnerability | 0.19 | 0.00 | 2149.83 |
| c | Continental | overlap | 0.20 | 0.00 | 2455.77 |
| z | Continental | overlap | -0.07 | 0.00 | -1503.03 |
| c | Continental | basal | 0.52 | 0.00 | 7449.76 |
| z | Continental | basal | 0.02 | 0.00 | 1396.51 |
| c | Continental | Top | 0.03 | 0.00 | 1410.32 |
| z | Continental | Top | -0.14 | 0.00 | -1692.71 |
| c | Continental | intermediate | 0.47 | 0.00 | 4963.60 |
| z | Continental | intermediate | -0.03 | 0.00 | -1246.94 |
| c | Mediterranean | species | 50.34 | 0.10 | 527.94 |
| z | Mediterranean | species | 0.29 | 0.00 | 1331.51 |
| c | Mediterranean | links | 92.96 | 0.47 | 196.96 |
| z | Mediterranean | links | 0.64 | 0.00 | 1132.57 |
| c | Mediterranean | links per sp | 3.26 | 0.01 | 458.52 |
| z | Mediterranean | links per sp | 0.29 | 0.00 | 1161.26 |
| c | Mediterranean | generality | 6.15 | 0.01 | 430.41 |
| z | Mediterranean | generality | 0.33 | 0.00 | 1267.16 |
| c | Mediterranean | vulnerability | 3.37 | 0.01 | 470.24 |
| z | Mediterranean | vulnerability | 0.29 | 0.00 | 1179.52 |
| c | Mediterranean | SD generality | 6.02 | 0.01 | 537.40 |
| z | Mediterranean | SD generality | 0.30 | 0.00 | 1413.49 |
| c | Mediterranean | SD vulnerability | 2.89 | 0.00 | 610.10 |
| z | Mediterranean | SD vulnerability | 0.28 | 0.00 | 1495.71 |
| c | Mediterranean | overlap | 0.18 | 0.00 | 2383.95 |
| z | Mediterranean | overlap | -0.07 | 0.00 | -1449.37 |
| c | Mediterranean | basal | 0.54 | 0.00 | 6974.35 |
| z | Mediterranean | basal | 0.02 | 0.00 | 1330.64 |
| c | Mediterranean | Top | 0.03 | 0.00 | 443.24 |
| z | Mediterranean | Top | -0.15 | 0.00 | -525.06 |
| c | Mediterranean | intermediate | 0.45 | 0.00 | 4092.08 |
| z | Mediterranean | intermediate | -0.03 | 0.00 | -1148.11 |
| c | Pannonian | species | 202.54 | 0.11 | 1900.83 |
| z | Pannonian | species | 0.08 | 0.00 | 946.36 |
| c | Pannonian | links | 2599.35 | 3.38 | 768.50 |
| z | Pannonian | links | 0.16 | 0.00 | 811.17 |
| c | Pannonian | links per sp | 13.02 | 0.01 | 1342.74 |
| z | Pannonian | links per sp | 0.08 | 0.00 | 709.28 |
| c | Pannonian | generality | 32.94 | 0.02 | 1595.18 |
| z | Pannonian | generality | 0.08 | 0.00 | 815.36 |
| c | Pannonian | vulnerability | 13.40 | 0.01 | 1399.61 |
| z | Pannonian | vulnerability | 0.08 | 0.00 | 713.57 |
| c | Pannonian | SD generality | 27.05 | 0.01 | 2226.92 |
| z | Pannonian | SD generality | 0.07 | 0.00 | 1046.70 |
| c | Pannonian | SD vulnerability | 12.68 | 0.01 | 1955.37 |
| z | Pannonian | SD vulnerability | 0.07 | 0.00 | 890.67 |
| c | Pannonian | overlap | 0.12 | 0.00 | 1659.46 |
| z | Pannonian | overlap | 0.01 | 0.00 | 94.93 |
| c | Pannonian | basal | 0.60 | 0.00 | 5063.52 |
| z | Pannonian | basal | 0.00 | 0.00 | -57.98 |
| c | Pannonian | Top | 0.05 | 0.00 | 246.99 |
| z | Pannonian | Top | -0.23 | 0.00 | -334.51 |
| c | Pannonian | intermediate | 0.36 | 0.00 | 2630.91 |
| z | Pannonian | intermediate | 0.01 | 0.00 | 204.55 |
| c | Steppic | species | 58.42 | 0.09 | 660.96 |
| z | Steppic | species | 0.24 | 0.00 | 1406.98 |
| c | Steppic | links | 127.96 | 0.52 | 246.04 |
| z | Steppic | links | 0.54 | 0.00 | 1196.74 |
| c | Steppic | links per sp | 4.54 | 0.01 | 604.21 |
| z | Steppic | links per sp | 0.22 | 0.00 | 1147.99 |
| c | Steppic | generality | 6.70 | 0.01 | 458.81 |
| z | Steppic | generality | 0.28 | 0.00 | 1158.95 |
| c | Steppic | vulnerability | 4.63 | 0.01 | 591.44 |
| z | Steppic | vulnerability | 0.21 | 0.00 | 1119.19 |
| c | Steppic | SD generality | 8.42 | 0.01 | 799.80 |
| z | Steppic | SD generality | 0.23 | 0.00 | 1619.28 |
| c | Steppic | SD vulnerability | 4.14 | 0.01 | 742.37 |
| z | Steppic | SD vulnerability | 0.21 | 0.00 | 1349.51 |
| c | Steppic | overlap | 0.23 | 0.00 | 1943.15 |
| z | Steppic | overlap | -0.09 | 0.00 | -1507.09 |
| c | Steppic | basal | 0.49 | 0.00 | 6188.47 |
| z | Steppic | basal | 0.03 | 0.00 | 1528.13 |
| c | Steppic | Top | 0.01 | 0.00 | 549.72 |
| z | Steppic | Top | -0.04 | 0.00 | -208.45 |
| c | Steppic | intermediate | 0.52 | 0.00 | 3653.49 |
| z | Steppic | intermediate | -0.04 | 0.00 | -1291.04 |

**Appendix S4.** Relationships of network properties with species richness.

Linear model results for the correlations of all network properties with species richness at each biogeographical region. Estimates, T values and adjusted-R^2^ are provided for each correlation.

|  | | Region | Property | Estimate | Std. Error | t value | Adjusted-R^2^ |
| --- | --- | --- | --- | --- | --- | --- | --- |
| log10(species) | Alpine | log10(links) | 1.85 | 0.00 | 10072.70 | 0.99 |  |
| log10(species) | Anatolian | log10(links) | 1.84 | 0.00 | 8154.93 | 1.00 |  |
| log10(species) | Arctic | log10(links) | 1.75 | 0.00 | 5231.67 | 0.99 |  |
| log10(species) | Atlantic | log10(links) | 2.05 | 0.00 | 6711.62 | 0.99 |  |
| log10(species) | BlackSea | log10(links) | 1.79 | 0.00 | 2597.88 | 0.99 |  |
| log10(species) | Boreal | log10(links) | 1.60 | 0.00 | 16356.72 | 0.99 |  |
| log10(species) | Continental | log10(links) | 1.88 | 0.00 | 14389.49 | 0.99 |  |
| log10(species) | Mediterranean | log10(links) | 2.00 | 0.00 | 12027.21 | 1.00 |  |
| log10(species) | Pannonian | log10(links) | 2.08 | 0.00 | 2708.74 | 0.99 |  |
| log10(species) | Steppic | log10(links) | 1.91 | 0.00 | 11793.12 | 0.99 |  |
| species | | Alpine | links per sp | 0.06 | 0.00 | 4635.26 | 0.97 |
| species | | Anatolian | links per sp | 0.06 | 0.00 | 4086.80 | 0.98 |
| species | | Arctic | links per sp | 0.06 | 0.00 | 1973.02 | 0.92 |
| species | | Atlantic | links per sp | 0.06 | 0.00 | 3854.87 | 0.97 |
| species | | BlackSea | links per sp | 0.06 | 0.00 | 1101.74 | 0.94 |
| species | | Boreal | links per sp | 0.04 | 0.00 | 6228.28 | 0.96 |
| species | | Continental | links per sp | 0.06 | 0.00 | 6705.26 | 0.97 |
| species | | Mediterranean | links per sp | 0.07 | 0.00 | 7570.58 | 0.99 |
| species | | Pannonian | links per sp | 0.07 | 0.00 | 1454.16 | 0.96 |
| species | | Steppic | links per sp | 0.06 | 0.00 | 6265.62 | 0.98 |
| species | | Alpine | generality | 0.18 | 0.00 | 4522.76 | 0.97 |
| species | | Anatolian | generality | 0.20 | 0.00 | 3204.82 | 0.98 |
| species | | Arctic | generality | 0.14 | 0.00 | 1853.78 | 0.91 |
| species | | Atlantic | generality | 0.17 | 0.00 | 4176.65 | 0.97 |
| species | | BlackSea | generality | 0.15 | 0.00 | 964.44 | 0.92 |
| species | | Boreal | generality | 0.10 | 0.00 | 3137.42 | 0.85 |
| species | | Continental | generality | 0.18 | 0.00 | 7902.40 | 0.98 |
| species | | Mediterranean | generality | 0.21 | 0.00 | 9663.55 | 0.99 |
| species | | Pannonian | generality | 0.17 | 0.00 | 1289.39 | 0.95 |
| species | | Steppic | generality | 0.20 | 0.00 | 6106.53 | 0.98 |
| species | | Alpine | vulnerability | 0.06 | 0.00 | 4690.57 | 0.97 |
| species | | Anatolian | vulnerability | 0.06 | 0.00 | 3738.19 | 0.98 |
| species | | Arctic | vulnerability | 0.06 | 0.00 | 1738.77 | 0.90 |
| species | | Atlantic | vulnerability | 0.06 | 0.00 | 3809.60 | 0.97 |
| species | | BlackSea | vulnerability | 0.06 | 0.00 | 976.57 | 0.92 |
| species | | Boreal | vulnerability | 0.04 | 0.00 | 6159.13 | 0.96 |
| species | | Continental | vulnerability | 0.06 | 0.00 | 6655.23 | 0.97 |
| species | | Mediterranean | vulnerability | 0.07 | 0.00 | 7830.15 | 0.99 |
| species | | Pannonian | vulnerability | 0.07 | 0.00 | 1450.81 | 0.96 |
| species | | Steppic | vulnerability | 0.06 | 0.00 | 5708.38 | 0.98 |
| species | | Alpine | top | -9E-06 | 1E-08 | -623.49 | 0.41 |
| species | | Anatolian | top | -5E-05 | 1E-07 | -377.46 | 0.35 |
| species | | Arctic | top | -2E-04 | 3E-07 | -590.92 | 0.51 |
| species | | Atlantic | top | -6E-05 | 1E-07 | -582.11 | 0.40 |
| species | | BlackSea | top | -6E-05 | 3E-07 | -217.94 | 0.36 |
| species | | Boreal | top | -7E-06 | 2E-08 | -369.39 | 0.07 |
| species | | Continental | top | -2E-05 | 8E-09 | -2064.19 | 0.74 |
| species | | Mediterranean | top | -1E-05 | 2E-08 | -624.26 | 0.35 |
| species | | Pannonian | top | -1E-04 | 7E-07 | -218.22 | 0.35 |
| species | | Steppic | top | -2E-06 | 2E-08 | -105.93 | 0.01 |
| species | | Alpine | intermediate | -7E-05 | 1E-07 | -630.77 | 0.41 |
| species | | Anatolian | intermediate | -1E-04 | 3E-07 | -371.57 | 0.34 |
| species | | Arctic | intermediate | 6E-04 | 6E-07 | 1057.88 | 0.77 |
| species | | Atlantic | intermediate | 6E-05 | 2E-07 | 332.27 | 0.18 |
| species | | BlackSea | intermediate | 7E-05 | 5E-07 | 138.04 | 0.19 |
| species | | Boreal | intermediate | 7E-05 | 2E-07 | 369.31 | 0.07 |
| species | | Continental | intermediate | -1E-04 | 7E-08 | -2108.13 | 0.74 |
| species | | Mediterranean | intermediate | -8E-05 | 1E-07 | -778.00 | 0.46 |
| species | | Pannonian | intermediate | 2E-04 | 1E-06 | 209.80 | 0.33 |
| species | | Steppic | intermediate | -2E-04 | 1E-07 | -2021.85 | 0.84 |
| species | | Alpine | basal | 8E-05 | 1E-07 | 714.93 | 0.47 |
| species | | Anatolian | basal | 2E-04 | 3E-07 | 620.16 | 0.59 |
| species | | Arctic | basal | -4E-04 | 7E-07 | -635.08 | 0.54 |
| species | | Atlantic | basal | 3E-06 | 2E-07 | 20.12 | 0.00 |
| species | | BlackSea | basal | 2E-05 | 4E-07 | 39.47 | 0.02 |
| species | | Boreal | basal | -6E-05 | 2E-07 | -304.86 | 0.05 |
| species | | Continental | basal | 2E-04 | 7E-08 | 2348.63 | 0.78 |
| species | | Mediterranean | basal | 1E-04 | 9E-08 | 1136.23 | 0.64 |
| species | | Pannonian | basal | -7E-05 | 9E-07 | -73.29 | 0.06 |
| species | | Steppic | basal | 2E-04 | 1E-07 | 1917.09 | 0.82 |
| species | | Alpine | SD generality | 0.12 | 0.00 | 7962.19 | 0.99 |
| species | | Anatolian | SD generality | 0.14 | 0.00 | 7366.41 | 0.99 |
| species | | Arctic | SD generality | 0.13 | 0.00 | 4005.63 | 0.97 |
| species | | Atlantic | SD generality | 0.12 | 0.00 | 7719.24 | 0.99 |
| species | | BlackSea | SD generality | 0.12 | 0.00 | 1872.95 | 0.97 |
| species | | Boreal | SD generality | 0.09 | 0.00 | 7530.21 | 0.96 |
| species | | Continental | SD generality | 0.12 | 0.00 | 14474.78 | 0.99 |
| species | | Mediterranean | SD generality | 0.14 | 0.00 | 10773.03 | 0.99 |
| species | | Pannonian | SD generality | 0.12 | 0.00 | 1853.36 | 0.96 |
| species | | Steppic | SD generality | 0.13 | 0.00 | 9410.13 | 0.99 |
| species | | Alpine | SD vulnerability | 0.05 | 0.00 | 4223.76 | 0.95 |
| species | | Anatolian | SD vulnerability | 0.05 | 0.00 | 4277.17 | 0.98 |
| species | | Arctic | SD vulnerability | 0.04 | 0.00 | 2401.98 | 0.92 |
| species | | Atlantic | SD vulnerability | 0.06 | 0.00 | 3188.08 | 0.93 |
| species | | BlackSea | SD vulnerability | 0.05 | 0.00 | 1743.70 | 0.96 |
| species | | Boreal | SD vulnerability | 0.04 | 0.00 | 5020.82 | 0.91 |
| species | | Continental | SD vulnerability | 0.05 | 0.00 | 7460.66 | 0.96 |
| species | | Mediterranean | SD vulnerability | 0.05 | 0.00 | 10284.56 | 0.99 |
| species | | Pannonian | SD vulnerability | 0.06 | 0.00 | 1768.15 | 0.96 |
| species | | Steppic | SD vulnerability | 0.05 | 0.00 | 6226.19 | 0.97 |
| species | | Alpine | overlap | -5E-05 | 4E-08 | -1E+03 | 0.60 |
| species | | Anatolian | overlap | -1E-04 | 1E-07 | -8E+02 | 0.59 |
| species | | Arctic | overlap | 2E-04 | 2E-07 | 8E+02 | 0.54 |
| species | | Atlantic | overlap | 3E-06 | 8E-08 | 4E+01 | 0.00 |
| species | | BlackSea | overlap | -2E-05 | 2E-07 | -1E+02 | 0.07 |
| species | | Boreal | overlap | -3E-05 | 8E-08 | -3E+02 | 0.04 |
| species | | Continental | overlap | -1E-04 | 3E-08 | -3E+03 | 0.83 |
| species | | Mediterranean | overlap | -6E-05 | 3E-08 | -2E+03 | 0.75 |
| species | | Pannonian | overlap | 7E-05 | 5E-07 | 1E+02 | 0.13 |
| species | | Steppic | overlap | -1E-04 | 5E-08 | -3E+03 | 0.85 |

**Appendix S5.** Different increase in species richness across trophic levels.

Relationship between the number of species in each trophic level with the total number of species in each biogeographical region in Europe. Dotted line, basal species; black line, intermediate species; dashed line, top species. Shaded areas correspond to 95% confidence intervals.


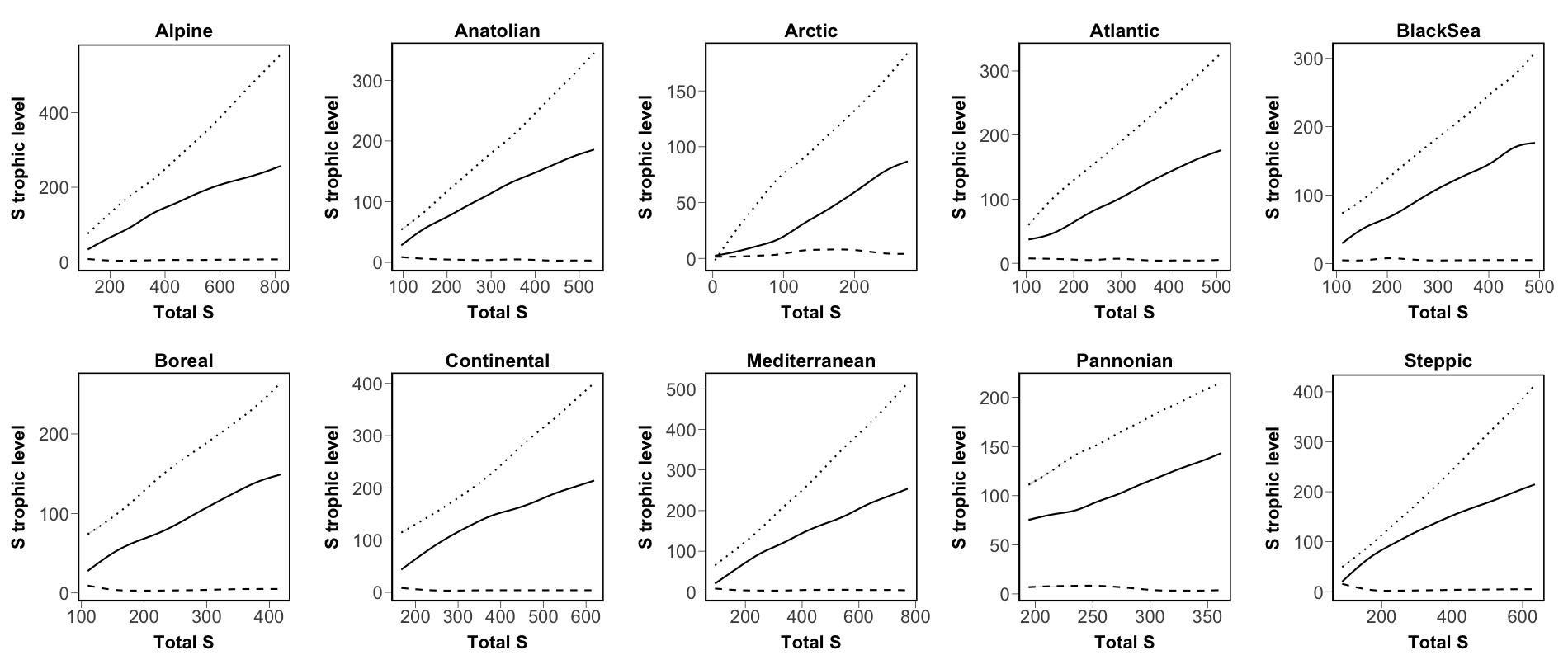


Despite the differences in the accumulation of species across trophic levels, the proportions are kept constant. Taking the Alpine region as an example, where the number of basal species increases faster that intermediate and top (being top the slowest), if we look at three region of the richness range (orange: small scales, pink: medium scales, blue: large scales) we can observe how the proportion of species level is constant even if the number of species of each trophic level is changing differently. For each region of the richness range, mean number of species at each trophic level across the range considered are reported.

**Appendix S6.** Species-Area relationship across biogeographical regions in Europe.

Relationship between the number of species with area in each biogeographical region in Europe. Black dots correspond to data points where the 100 replications are shown and red line correspond to the predicted values from the power function fit.


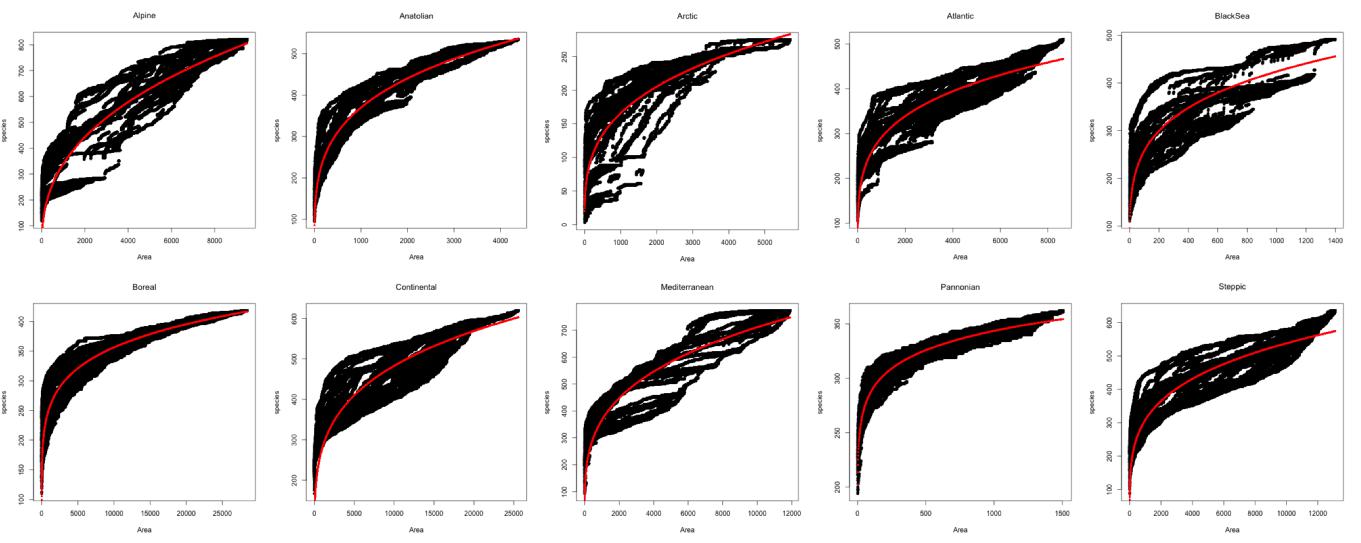


**Appendix S7.** Contribution of each biogeographical region to the global species-area relationship in Europe. Relationship between the number of species with area across all the European range considering the contribution of each biogeographical region. Notice that for the realization of this figure we used a linear neighbouring aggregation following the north-south gradient instead of the method used in the rest of the paper. Each coloured line represent the end of all the cells corresponding to a given biogeographical region. Therefore, the increase in the number of species from one region to the next correspond to the sampling of new species not found in the previous region. The pattern observed here changes when following the opposite latitudinal gradient. That is, starting with the species rich regions, such as Mediterranean.


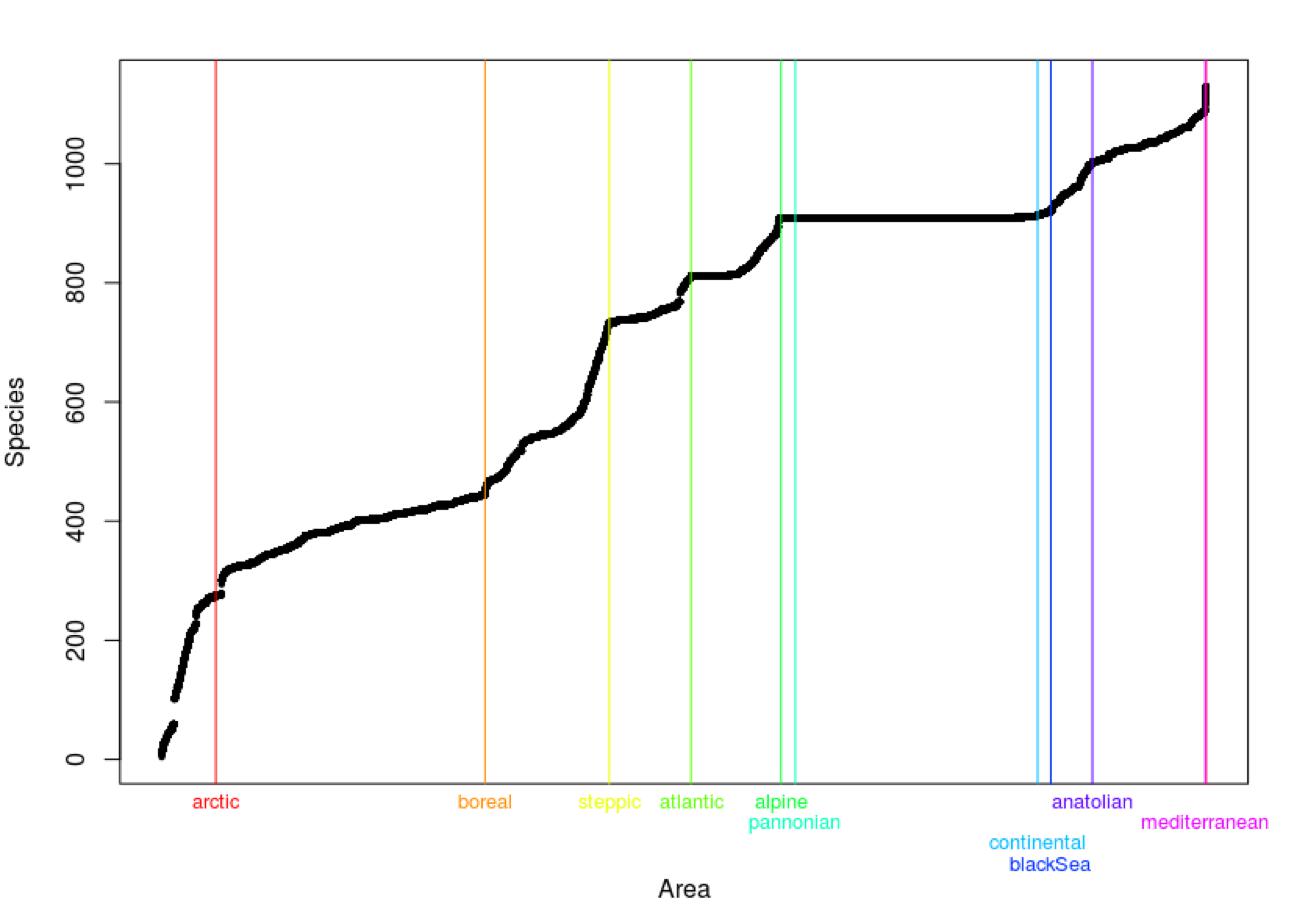


**Appendix S8.** Network-Area relationships across biogeographical regions in Europe.

Relationship between the number of species, links, links per species, generality, vulnerability (and their standard deviations), fraction of consumer’s diet overlap and fraction of species per trophic level with area in each biogeographical region in Europe. Different colours represent data points for each bioregions where the 5 replications are shown to facilitate visualisation.

**Appendix S9.** Null model results to test the contribution of species richness on the spatial scaling of network structure.

The figures below illustrate the results of the network-area relationships built with the null model-1 and the tables report the results of the power function fits. Notice that the plots show extremely similar patterns than those shown in Figure 2 and 3 (except for consumer’s overlap, which is much higher in the networks built with the null model), and that the differences between the estimates parameters reported in the table and those shown in Appendix S3 are <0.1 for all bioregions and properties.

Power function fit for all network properties built with the null model for each biogeographical region (100 replicates per bioregion). Fit of each network property of each biogeographical region to a power function (f(x) = cx^z^), where c is the scaling factor and z is the exponent parameter that determines the function’s rates of growth and its overall shape. We used nonlinear least squares (NLS) with the ’nls’ function in R.

| Parameters | Dataset | Property | Estimate | Std. Error | t value |
| --- | --- | --- | --- | --- | --- |
| c | Alpine | species | 25.88 | 0.04 | 692.87 |
| z | Alpine | species | 0.38 | 0.00 | 2242.93 |
| c | Alpine | links | 26.89 | 0.08 | 318.46 |
| z | Alpine | links | 0.80 | 0.00 | 2246.20 |
| c | Alpine | links per sp | 1.59 | 0.00 | 665.57 |
| z | Alpine | links per sp | 0.38 | 0.00 | 2157.39 |
| c | Alpine | generality | 5.31 | 0.01 | 689.51 |
| z | Alpine | generality | 0.37 | 0.00 | 2180.10 |
| c | Alpine | vulnerability | 1.81 | 0.00 | 690.57 |
| z | Alpine | vulnerability | 0.36 | 0.00 | 2155.93 |
| c | Alpine | SD generality | 3.35 | 0.00 | 678.46 |
| z | Alpine | SD generality | 0.38 | 0.00 | 2199.74 |
| c | Alpine | SD vulnerability | 1.33 | 0.00 | 673.22 |
| z | Alpine | SD vulnerability | 0.37 | 0.00 | 2155.61 |
| c | Alpine | basals | 0.66 | 0.00 | 8541.69 |
| z | Alpine | basals | 0.00 | 0.00 | 220.56 |
| c | Alpine | tops | 0.12 | 0.00 | 488.96 |
| z | Alpine | tops | -0.24 | 0.00 | -874.89 |
| c | Alpine | intermediates | 0.23 | 0.00 | 2706.02 |
| z | Alpine | intermediates | 0.03 | 0.00 | 713.59 |
| c | Alpine | overlaps | 0.72 | 0.00 | 6022.65 |
| z | Alpine | overlaps | 0.00 | 0.00 | 121.89 |
| c | Anatolian | species | 64.11 | 0.04 | 1459.71 |
| z | Anatolian | species | 0.25 | 0.00 | 2863.02 |
| c | Anatolian | links | 260.20 | 0.43 | 607.53 |
| z | Anatolian | links | 0.52 | 0.00 | 2507.41 |
| c | Anatolian | links per sp | 4.59 | 0.00 | 1177.05 |
| z | Anatolian | links per sp | 0.25 | 0.00 | 2304.37 |
| c | Anatolian | generality | 13.48 | 0.01 | 1261.91 |
| z | Anatolian | generality | 0.25 | 0.00 | 2410.66 |
| c | Anatolian | vulnerability | 4.93 | 0.00 | 1212.94 |
| z | Anatolian | vulnerability | 0.24 | 0.00 | 2297.55 |
| c | Anatolian | SD generality | 9.50 | 0.01 | 1292.02 |
| z | Anatolian | SD generality | 0.25 | 0.00 | 2534.67 |
| c | Anatolian | SD vulnerability | 3.83 | 0.00 | 1222.51 |
| z | Anatolian | SD vulnerability | 0.25 | 0.00 | 2363.52 |
| c | Anatolian | basals | 0.63 | 0.00 | 6059.40 |
| z | Anatolian | basals | 0.00 | 0.00 | 111.09 |
| c | Anatolian | tops | 0.07 | 0.00 | 341.50 |
| z | Anatolian | tops | -0.29 | 0.00 | -640.07 |
| c | Anatolian | intermediates | 0.30 | 0.00 | 2691.52 |
| z | Anatolian | intermediates | 0.02 | 0.00 | 413.43 |
| c | Anatolian | overlaps | 0.72 | 0.00 | 4609.58 |
| z | Anatolian | overlaps | 0.00 | 0.00 | 25.68 |
| c | Arctic | species | 17.19 | 0.03 | 633.46 |
| z | Arctic | species | 0.32 | 0.00 | 1658.49 |
| c | Arctic | links | 35.04 | 0.09 | 394.12 |
| z | Arctic | links | 0.59 | 0.00 | 1897.47 |
| c | Arctic | links per sp | 1.23 | 0.00 | 585.04 |
| z | Arctic | links per sp | 0.32 | 0.00 | 1526.04 |
| c | Arctic | generality | 5.24 | 0.01 | 658.32 |
| z | Arctic | generality | 0.28 | 0.00 | 1495.20 |
| c | Arctic | vulnerability | 1.38 | 0.00 | 607.94 |
| z | Arctic | vulnerability | 0.31 | 0.00 | 1524.80 |
| c | Arctic | SD generality | 2.69 | 0.00 | 613.28 |
| z | Arctic | SD generality | 0.32 | 0.00 | 1606.32 |
| c | Arctic | SD vulnerability | 0.85 | 0.00 | 604.53 |
| z | Arctic | SD vulnerability | 0.31 | 0.00 | 1503.48 |
| c | Arctic | basals | 0.73 | 0.00 | 3305.07 |
| z | Arctic | basals | -0.01 | 0.00 | -234.06 |
| c | Arctic | tops | 0.23 | 0.00 | 430.25 |
| z | Arctic | tops | -0.29 | 0.00 | -835.64 |
| c | Arctic | intermediates | 0.12 | 0.00 | 876.37 |
| z | Arctic | intermediates | 0.11 | 0.00 | 766.49 |
| c | Arctic | overlaps | 0.82 | 0.00 | 2671.77 |
| z | Arctic | overlaps | 0.00 | 0.00 | -98.28 |
| c | Atlantic | species | 65.92 | 0.06 | 1177.59 |
| z | Atlantic | species | 0.22 | 0.00 | 2141.70 |
| c | Atlantic | links | 167.23 | 0.35 | 477.42 |
| z | Atlantic | links | 0.49 | 0.00 | 1992.91 |
| c | Atlantic | links per sp | 3.97 | 0.00 | 1051.25 |
| z | Atlantic | links per sp | 0.22 | 0.00 | 1911.94 |
| c | Atlantic | generality | 11.64 | 0.01 | 1111.63 |
| z | Atlantic | generality | 0.21 | 0.00 | 1975.98 |
| c | Atlantic | vulnerability | 4.31 | 0.00 | 1089.48 |
| z | Atlantic | vulnerability | 0.21 | 0.00 | 1906.75 |
| c | Atlantic | SD generality | 8.26 | 0.01 | 1103.59 |
| z | Atlantic | SD generality | 0.22 | 0.00 | 2011.39 |
| c | Atlantic | SD vulnerability | 3.44 | 0.00 | 1077.07 |
| z | Atlantic | SD vulnerability | 0.21 | 0.00 | 1918.55 |
| c | Atlantic | basals | 0.64 | 0.00 | 7698.30 |
| z | Atlantic | basals | 0.00 | 0.00 | 62.46 |
| c | Atlantic | tops | 0.09 | 0.00 | 424.67 |
| z | Atlantic | tops | -0.21 | 0.00 | -673.93 |
| c | Atlantic | intermediates | 0.28 | 0.00 | 2839.85 |
| z | Atlantic | intermediates | 0.02 | 0.00 | 489.48 |
| c | Atlantic | overlaps | 0.76 | 0.00 | 6057.46 |
| z | Atlantic | overlaps | 0.00 | 0.00 | 135.59 |
| c | BlackSea | species | 77.43 | 0.18 | 440.69 |
| z | BlackSea | species | 0.25 | 0.00 | 722.82 |
| c | BlackSea | links | 283.34 | 1.62 | 175.39 |
| z | BlackSea | links | 0.55 | 0.00 | 654.10 |
| c | BlackSea | links per sp | 5.12 | 0.01 | 409.69 |
| z | BlackSea | links per sp | 0.25 | 0.00 | 675.24 |
| c | BlackSea | generality | 14.54 | 0.03 | 431.98 |
| z | BlackSea | generality | 0.24 | 0.00 | 686.86 |
| c | BlackSea | vulnerability | 5.72 | 0.01 | 432.76 |
| z | BlackSea | vulnerability | 0.24 | 0.00 | 673.35 |
| c | BlackSea | SD generality | 10.38 | 0.02 | 422.01 |
| z | BlackSea | SD generality | 0.25 | 0.00 | 694.35 |
| c | BlackSea | SD vulnerability | 4.47 | 0.01 | 418.86 |
| z | BlackSea | SD vulnerability | 0.25 | 0.00 | 680.19 |
| c | BlackSea | basals | 0.62 | 0.00 | 3346.34 |
| z | BlackSea | basals | 0.00 | 0.00 | 41.33 |
| c | BlackSea | tops | 0.08 | 0.00 | 203.69 |
| z | BlackSea | tops | -0.23 | 0.00 | -272.91 |
| c | BlackSea | intermediates | 0.29 | 0.00 | 1276.78 |
| z | BlackSea | intermediates | 0.03 | 0.00 | 246.05 |
| c | BlackSea | overlaps | 0.73 | 0.00 | 2798.92 |
| z | BlackSea | overlaps | 0.00 | 0.00 | 17.42 |
| c | Boreal | species | 95.48 | 0.02 | 4887.62 |
| z | Boreal | species | 0.14 | 0.00 | 6671.77 |
| c | Boreal | links | 504.72 | 0.24 | 2146.05 |
| z | Boreal | links | 0.30 | 0.00 | 6115.62 |
| c | Boreal | links per sp | 5.74 | 0.00 | 3612.88 |
| z | Boreal | links per sp | 0.14 | 0.00 | 4913.82 |
| c | Boreal | generality | 16.81 | 0.00 | 3904.63 |
| z | Boreal | generality | 0.14 | 0.00 | 5034.20 |
| c | Boreal | vulnerability | 6.07 | 0.00 | 3722.15 |
| z | Boreal | vulnerability | 0.14 | 0.00 | 4912.40 |
| c | Boreal | SD generality | 12.33 | 0.00 | 3970.20 |
| z | Boreal | SD generality | 0.14 | 0.00 | 5424.47 |
| c | Boreal | SD vulnerability | 4.65 | 0.00 | 3690.13 |
| z | Boreal | SD vulnerability | 0.14 | 0.00 | 4914.33 |
| c | Boreal | basals | 0.66 | 0.00 | 15397.93 |
| z | Boreal | basals | 0.00 | 0.00 | -529.61 |
| c | Boreal | tops | 0.08 | 0.00 | 759.22 |
| z | Boreal | tops | -0.18 | 0.00 | -1190.83 |
| c | Boreal | intermediates | 0.29 | 0.00 | 6136.62 |
| z | Boreal | intermediates | 0.02 | 0.00 | 1132.42 |
| c | Boreal | overlaps | 0.76 | 0.00 | 11433.75 |
| z | Boreal | overlaps | 0.00 | 0.00 | -35.83 |
| c | Continental | species | 67.65 | 0.04 | 1732.61 |
| z | Continental | species | 0.22 | 0.00 | 3547.32 |
| c | Continental | links | 190.23 | 0.25 | 758.29 |
| z | Continental | links | 0.47 | 0.00 | 3449.33 |
| c | Continental | links per sp | 4.10 | 0.00 | 1558.99 |
| z | Continental | links per sp | 0.21 | 0.00 | 3181.38 |
| c | Continental | generality | 12.28 | 0.01 | 1647.67 |
| z | Continental | generality | 0.21 | 0.00 | 3259.33 |
| c | Continental | vulnerability | 4.42 | 0.00 | 1605.00 |
| z | Continental | vulnerability | 0.21 | 0.00 | 3170.72 |
| c | Continental | SD generality | 8.44 | 0.01 | 1630.40 |
| z | Continental | SD generality | 0.22 | 0.00 | 3337.63 |
| c | Continental | SD vulnerability | 3.57 | 0.00 | 1591.34 |
| z | Continental | SD vulnerability | 0.21 | 0.00 | 3197.33 |
| c | Continental | basals | 0.65 | 0.00 | 11789.17 |
| z | Continental | basals | 0.00 | 0.00 | -100.30 |
| c | Continental | tops | 0.10 | 0.00 | 474.53 |
| z | Continental | tops | -0.23 | 0.00 | -932.05 |
| c | Continental | intermediates | 0.28 | 0.00 | 4228.97 |
| z | Continental | intermediates | 0.02 | 0.00 | 779.13 |
| c | Continental | overlaps | 0.74 | 0.00 | 8548.20 |
| z | Continental | overlaps | 0.00 | 0.00 | -23.84 |
| c | Mediterranean | species | 43.88 | 0.07 | 632.34 |
| z | Mediterranean | species | 0.30 | 0.00 | 1676.99 |
| c | Mediterranean | links | 70.91 | 0.28 | 249.75 |
| z | Mediterranean | links | 0.67 | 0.00 | 1501.90 |
| c | Mediterranean | links per sp | 2.90 | 0.00 | 603.42 |
| z | Mediterranean | links per sp | 0.30 | 0.00 | 1596.94 |
| c | Mediterranean | generality | 8.77 | 0.01 | 624.47 |
| z | Mediterranean | generality | 0.30 | 0.00 | 1624.79 |
| c | Mediterranean | vulnerability | 3.12 | 0.01 | 618.22 |
| z | Mediterranean | vulnerability | 0.29 | 0.00 | 1594.47 |
| c | Mediterranean | SD generality | 5.95 | 0.01 | 616.41 |
| z | Mediterranean | SD generality | 0.30 | 0.00 | 1635.22 |
| c | Mediterranean | SD vulnerability | 2.42 | 0.00 | 614.18 |
| z | Mediterranean | SD vulnerability | 0.30 | 0.00 | 1605.20 |
| c | Mediterranean | basals | 0.65 | 0.00 | 7231.97 |
| z | Mediterranean | basals | 0.00 | 0.00 | 143.24 |
| c | Mediterranean | tops | 0.08 | 0.00 | 335.22 |
| z | Mediterranean | tops | -0.26 | 0.00 | -664.88 |
| c | Mediterranean | intermediates | 0.28 | 0.00 | 2875.99 |
| z | Mediterranean | intermediates | 0.02 | 0.00 | 394.30 |
| c | Mediterranean | overlaps | 0.70 | 0.00 | 5001.34 |
| z | Mediterranean | overlaps | 0.00 | 0.00 | 99.65 |
| c | Pannonian | species | 204.36 | 0.08 | 2471.75 |
| z | Pannonian | species | 0.08 | 0.00 | 1215.13 |
| c | Pannonian | links | 2692.06 | 2.87 | 938.83 |
| z | Pannonian | links | 0.15 | 0.00 | 951.33 |
| c | Pannonian | links per sp | 13.43 | 0.01 | 1435.42 |
| z | Pannonian | links per sp | 0.08 | 0.00 | 700.55 |
| c | Pannonian | generality | 33.49 | 0.02 | 1570.80 |
| z | Pannonian | generality | 0.07 | 0.00 | 749.46 |
| c | Pannonian | vulnerability | 13.88 | 0.01 | 1484.78 |
| z | Pannonian | vulnerability | 0.07 | 0.00 | 696.36 |
| c | Pannonian | SD generality | 26.09 | 0.02 | 1630.69 |
| z | Pannonian | SD generality | 0.08 | 0.00 | 796.47 |
| c | Pannonian | SD vulnerability | 12.26 | 0.01 | 1474.06 |
| z | Pannonian | SD vulnerability | 0.07 | 0.00 | 711.81 |
| c | Pannonian | basals | 0.59 | 0.00 | 4201.13 |
| z | Pannonian | basals | 0.00 | 0.00 | 2.60 |
| c | Pannonian | tops | 0.03 | 0.00 | 153.64 |
| z | Pannonian | tops | -0.13 | 0.00 | -122.08 |
| c | Pannonian | intermediates | 0.37 | 0.00 | 2024.63 |
| z | Pannonian | intermediates | 0.01 | 0.00 | 96.70 |
| c | Pannonian | overlaps | 0.80 | 0.00 | 4479.66 |
| z | Pannonian | overlaps | 0.00 | 0.00 | -1.96 |
| c | Steppic | species | 56.87 | 0.07 | 866.63 |
| z | Steppic | species | 0.24 | 0.00 | 1871.73 |
| c | Steppic | links | 109.70 | 0.33 | 334.33 |
| z | Steppic | links | 0.56 | 0.00 | 1680.06 |
| c | Steppic | links per sp | 3.50 | 0.00 | 789.77 |
| z | Steppic | links per sp | 0.24 | 0.00 | 1708.41 |
| c | Steppic | generality | 10.94 | 0.01 | 829.62 |
| z | Steppic | generality | 0.24 | 0.00 | 1732.34 |
| c | Steppic | vulnerability | 3.92 | 0.00 | 823.18 |
| z | Steppic | vulnerability | 0.23 | 0.00 | 1701.86 |
| c | Steppic | SD generality | 7.37 | 0.01 | 822.55 |
| z | Steppic | SD generality | 0.24 | 0.00 | 1777.45 |
| c | Steppic | SD vulnerability | 2.98 | 0.00 | 807.48 |
| z | Steppic | SD vulnerability | 0.24 | 0.00 | 1722.55 |
| c | Steppic | basals | 0.64 | 0.00 | 6476.99 |
| z | Steppic | basals | 0.00 | 0.00 | 59.33 |
| c | Steppic | tops | 0.11 | 0.00 | 351.44 |
| z | Steppic | tops | -0.22 | 0.00 | -619.12 |
| c | Steppic | intermediates | 0.26 | 0.00 | 2265.28 |
| z | Steppic | intermediates | 0.03 | 0.00 | 526.61 |
| c | Steppic | overlaps | 0.73 | 0.00 | 4952.70 |
| z | Steppic | overlaps | 0.00 | 0.00 | 115.43 |

****Visual comparison between the z-exponent estimates of the power function fit for the original network-area relationships and those obtained with the null model. The x-axis shows the ratio between both z-exponents. Therefore, values close to 1 indicate that the z-exponent estimate obtained with the original networks is the same than the one obtained with the null model networks.

The figures below illustrate the results of the network-area relationships built with the null model-2, where the number of species and links are kept the same as in the original networks but the links are distributed randomly among the species breaking therefore the structure of the network. Notice that while for species, links, links per species, generality and vulnerability the spatial scaling is very similar to the original networks by construction, the standard deviation of generality and vulnerability show much smaller values given that all species have the same probability of having a link. Similarly, the percentages of species per trophic level are different from the original networks, indicating that they are inherited from the metaweb structure. Finally, the percentage of consumers’ diet overlap is much larger in the networks generated with the null model-2 and it increases with spatial scale, suggesting that in the original networks there might be spatial structuring of species that minimizes consumers’ diet overlap.

**
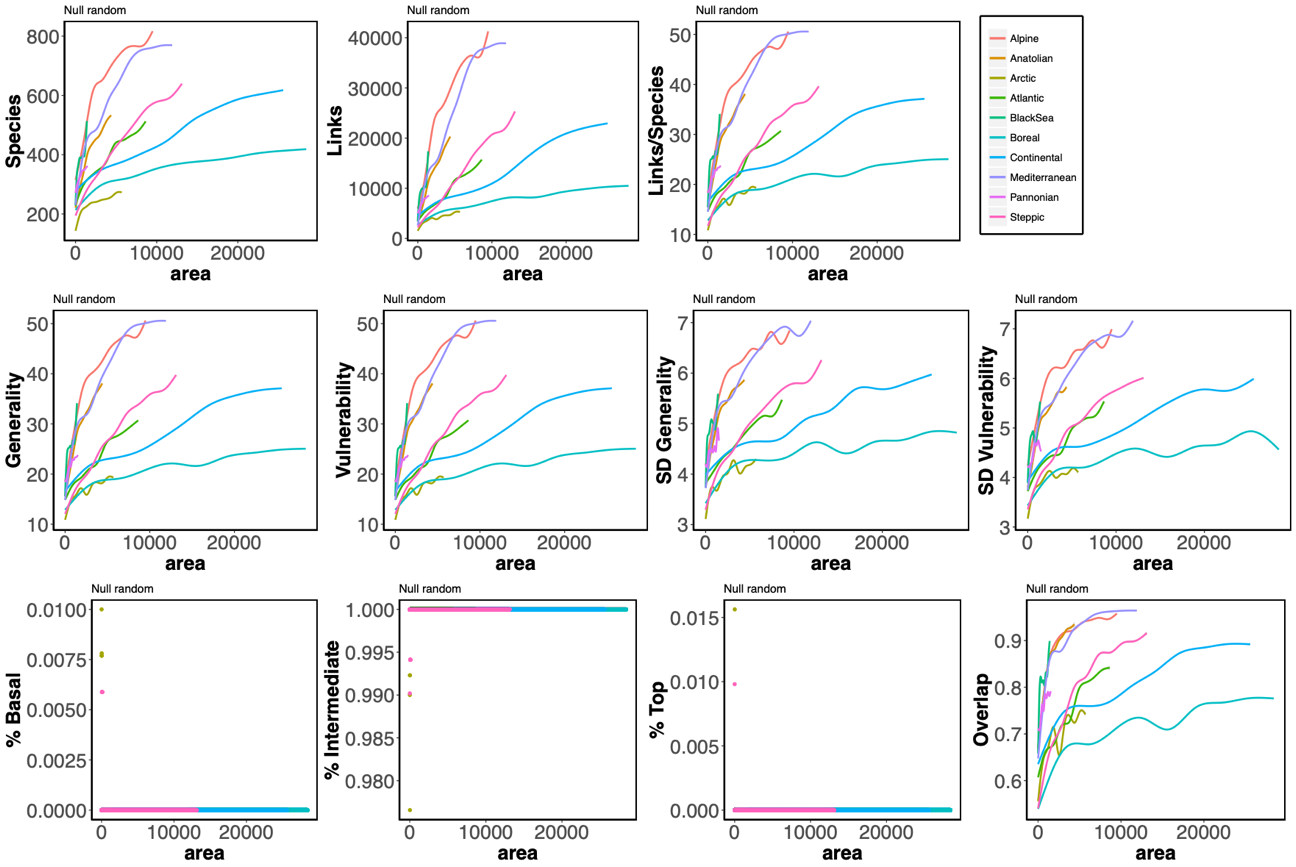
**

The figures below illustrate the relationship between the network properties normalised by the number of species with area for the original networks (first row), null model-1 (second row) and null model-3 (third row). Notice that when dividing each network property by the number of species present in the network, there is almost no variation across spatial scales for most bioregions (Arctic being the exception).

**References**

Prunier, J. G., M. Colyn, X. Legendre, K. F. Nimon, and M.-C. Flamand. 2015. Multicollinearity in spatial genetics: separating the wheat from the chaff using commonality analyses. Molecular ecology 24:263–283.
